# Mendelian randomization and multiomics comprehensively reveal the causal relationship and potential mechanism between atrial fibrillation and gastric cancer

**DOI:** 10.3389/fgene.2025.1446661

**Published:** 2025-02-03

**Authors:** Zhao Sicheng, Zhang Jingcheng, Zhang Shuo, Lou Jiaheng, Cai Yan, Bai Xing, Jiang Tao, Zhang Guangji

**Affiliations:** ^1^ School of Basic Medical Sciences, Zhejiang Chinese Medical University, Hangzhou, Zhejiang, China; ^2^ Key Laboratory of Blood-stasis-toxin Syndrome of Zhejiang Province, Hangzhou, Zhejiang, China; ^3^ Traditional Chinese Medicine “Preventing Disease” Wisdom Health Project Research, Nanning, Zhejiang, China

**Keywords:** atrial fibrillation, gastric cancer, genetic instrument, mendelian randomization, single-cell sequencing, fibroblasts

## Abstract

**Objective:**

Gastric cancer is a harmful disease, the comorbidity mechanism and causality relationship between this disease and other diseases are worth studying.

**Methods:**

Using a two-sample Mendelian Randomization method, this study revealed the potential causal effect of atrial fibrillation (AF) on gastric cancer (GC) risk by constructing a genetic instrument containing 136 AF associated SNPs. Subsequently, analysis identifies 62 AF-GC co-associated genes and constructs a protein-protein interaction network of key genes. High-throughput sequencing data were further used to analyze the association between the two and their impact on the survival outcome of gastric cancer.

**Results:**

The results showed that AF was negatively associated with gastric cancer, and further analysis revealed that this relationship was independent of GC risk factors such as chronic gastritis, *Helicobacter pylori* infection, and alcohol consumption. Enrichment analysis reveals associations of key genes with pathways related to cardiovascular disease, inflammatory gastrointestinal diseases, and tumorigenesis. Through single-cell sequencing data analysis, fibroblast subpopulations associated with the key gene set are identified in GC, showing significant correlations with cancer progression and inflammation regulation pathways. Transcription factor analysis and developmental trajectory analysis reveal the potential role of fibroblasts in GC development. Finally, prognosis analysis and gene mutation analysis using TCGA-STAD data indicate an adverse prognosis associated with the key gene set in GC.

**Conclusion:**

This study provides new insights into the association between AF and GC and offers novel clues for understanding its impact on the pathogenesis and therapeutic strategies of GC.

## Introduction

Gastric cancer (GC) ranks as the fifth most common cancer globally and is the fourth leading cause of cancer-related deaths (Sung et al., 2021). Despite significant progress in the diagnosis and treatment of GC, there remain many areas in this field that require further exploration (Smyth et al., 2020). GC is a complex disease influenced by various factors, including environmental and genetic factors. Among these factors, *Helicobacter pylori* infection is considered a primary pathogen. Additionally, due to the stomach’s central role in the digestive process, dietary factors play a crucial role in GC occurrence. Known risk factors include chronic gastritis, alcohol consumption, low intake of fresh fruits and vegetables, and excessive consumption of pickled and smoked foods (Van Cutsem et al., 2016). Furthermore, research has focused on exploring the relationship between GC and other diseases, such as cardiovascular diseases, which has become a research focus.

Atrial fibrillation (AF) is a common cardiac arrhythmia, affecting approximately 10% of the population (Krijthe et al., 2013; Joseph et al., 2020). Early studies on AF often focused on its potential to trigger cardiac and vascular thrombosis. Contemporary research has revealed a potential connection between AF and cancer incidence, with newly diagnosed AF patients found to have a 41% increased risk of developing malignant tumors compared to the general population (Joseph et al., 2020). However, the relationship between AF and GC remains uncertain, primarily due to a lack of further stratified analysis and unclear temporal relationships. Therefore, our focus is on investigating AF, a common cardiac arrhythmia, its impact on GC occurrence, and exploring the related mechanisms.

**FIGURE 1:**
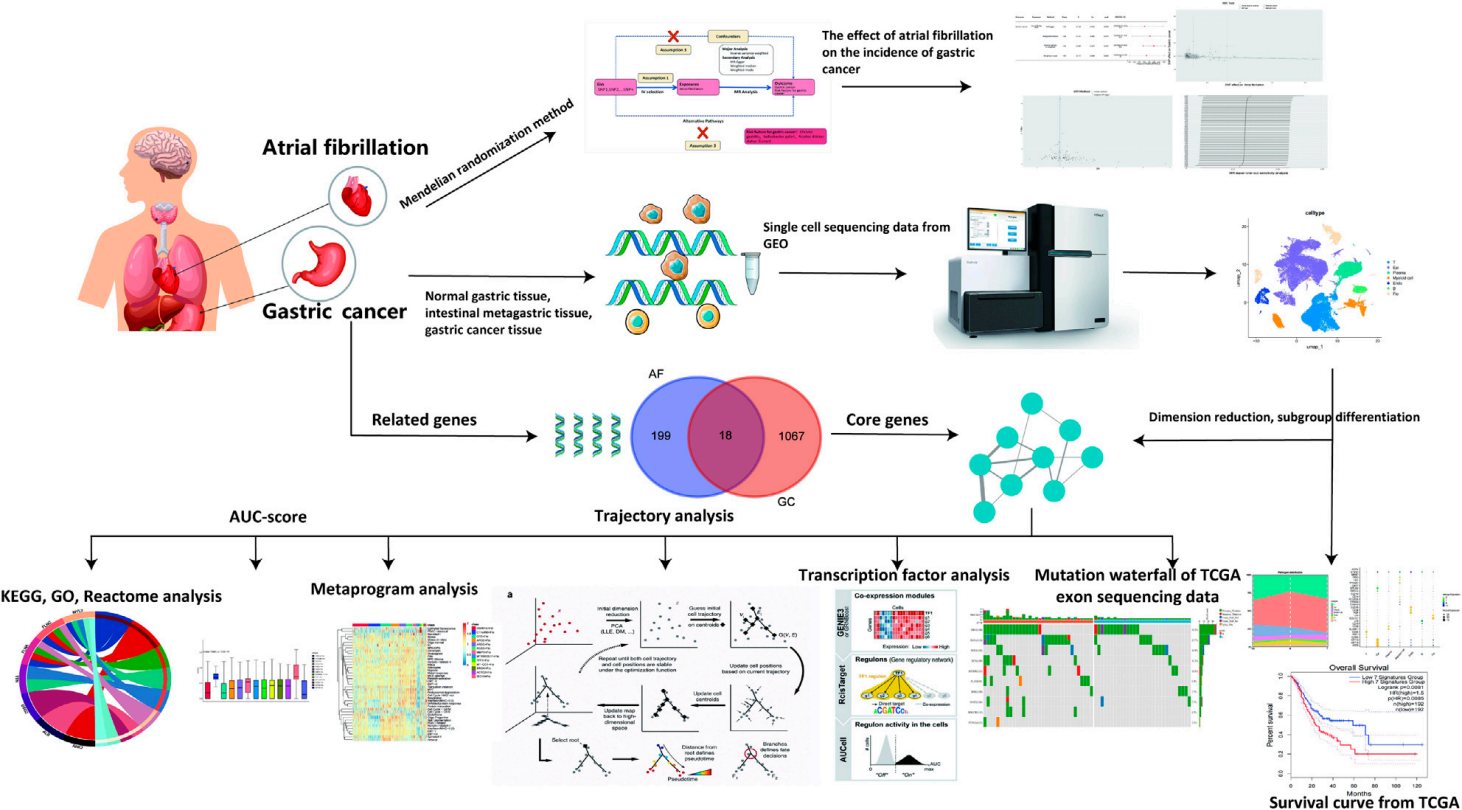
Research workflow.

Traditional observational studies face inherent challenges, including potential biases from confounding variables and reverse causality. Establishing a causal relationship between AF and GC using traditional clinical research methods is challenging. Mendelian Randomization studies can eliminate the influence of confounding factors and provide new insights into AF as a risk factor for GC development (Smith and Ebrahim, 2003; Smith and Ebrahim, 2004; Haaland et al., 2017). Meanwhile, high-throughput sequencing data link clinical phenomena with mechanisms. Through GC-related single-cell transcriptome sequencing data, potential mechanisms of how AF-related genes affect GC can be further explored. The research process is illustrated in Figure 1.

## Methods

### Data source for mendelian randomization study

**FIGURE 2:**
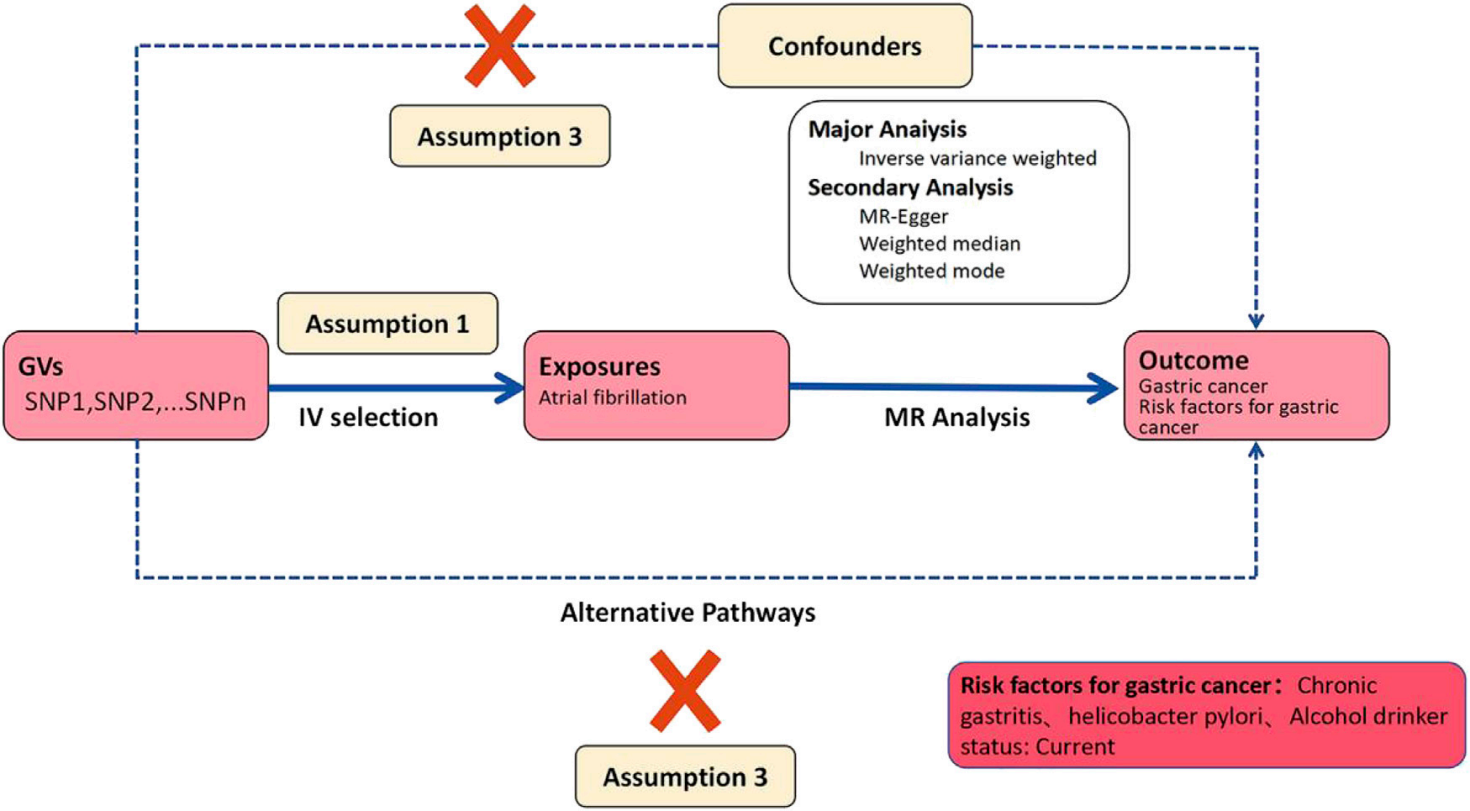
Workflow of two-sample mendelian randomization study.

This study constitutes an analysis of previously collected and publicly available data, including statistical summaries related to AF, GC, *Helicobacter pylori* infection, and alcohol consumption from large-scale public Genome-Wide Association Studies (GWAS), as detailed in Table 1. Due to the source and nature of the data, no additional ethical review or informed consent was required for this study. We employed a two-sample Mendelian Randomization analysis to assess the causal relationship between AF and GC. We chose AF as the exposure factor and GC as the outcome indicator. Additionally, we conducted two-sample Mendelian Randomization analyses using AF as the exposure factor and *Helicobacter pylori* infection, as well as alcohol consumption status, as the outcome indicators to examine whether AF affects GC through intermediary factors. The Mendelian Randomization study design process, as constructed in this paper, is illustrated in Figure 2.

**TABLE 1:** Mendelian randomized data source summary.

Phenotype	Type of trait	Author, published year	Consortium	Sample size	No. of cases (binary trait)	PMID
Atrial fibrillation	Binary	Roselli C et al., 2018	NA	537,409	55,114	29,892,015
Gastric cancer	Binary	Ishigaki K et al., 2019	NA	202,308	6,563	NA
Chronic gastritis	Binary	Ben Elsworth et al., 2018	MRC-IEU	463,010	1,467	NA
*helicobacter pylori*	Binary	Ben Elsworth et al., 2019	MRC-IEU	462,933	1,329	NA
Alcohol drinker status: Current	Binary	Neale lab et al., 2018	NA	360,726	336,919	NA

### Selection of genetic instrument variables

This study strictly adhered to quality control procedures. Initially, we selected Genome-Wide Association Studies (GWAS) data with deterministic correlations and filtered SNP loci with genome-wide significance (p < 5 × 10–6) for amalgamation. Subsequently, to mitigate the influence of linkage disequilibrium (LD) on the results, we conducted a clustering process by setting a parameter threshold (r2 < 0.001) to assess LD between SNPs, ensuring independence. The instrumental variables (IVs) used as exposure factors needed to satisfy three fundamental assumptions, the fulfillment of which enhances the IV’s testability and estimation accuracy: (1) the relevance assumption: genetic variants are associated with the exposure; (2) the independence assumption: genetic variants are unrelated to confounders between exposure and outcome; (3) the exclusion-restriction assumption: genetic variants affect the outcome solely through the exposure (Lawlor, 2016). Subsequently, summary statistics of qualified SNPs were extracted from the outcome GWAS. Finally, we ensured that the SNPs included in the dataset met the requirements of instrumental variables. Palindromic sequences were excluded to ensure that the influence of SNPs on exposure and outcome stemmed from the same allele. This series of steps ultimately identified the SNPs serving as genetic IVs in this study.

### Statistical analysis of mendelian randomization

After harmonizing the Genome-Wide Association Studies (GWAS) effect alleles for AF, GC, *Helicobacter pylori* (HP) infection, and alcohol consumption status, four Mendelian Randomization methods were selected. These methods include the Inverse Variance Weighted (IVW) test, Weighted Median Estimation, MR-Egger regression, and the Weighted Mode Estimation (WME). These methods were employed to evaluate the causal relationship between AF and GC risk as well as risk factors. The primary analytical method used was IVW, while WME and Cochran’s Q test were utilized to estimate heterogeneity in the causal effects of individual genetic variants. If level-specific effects or heterogeneity were detected, the fixed-effect IVW analysis should be chosen; conversely, random-effect IVW analysis should be employed (Burgess et al., 2013; Bowden et al., 2018). The IVW method does not consider the presence of intercept terms and utilizes the variance of the outcomes as fitting weights. Conversely, this method corrects for pleiotropic biases and detects directional pleiotropy but is susceptible to instrumental variable assumptions. When the Egger intercept of the linear regression approaches zero, it indicates the absence of directional pleiotropy, thus satisfying the exclusion-restriction assumption. The Weighted Median method combines data from multiple genetic variants into a single causal estimate and requires over 50% of the weight to come from valid instrumental variables to obtain reliable estimates of causal effects (Bowden et al., 2016). To ensure the reliability of Mendelian Randomization estimates, we also detected outliers that may affect Mendelian Randomization estimates by examining forest plots, funnel plots, scatter plots, and leave-one-out analysis.

To test the first assumption of relevance, we also used the F statistic (F = β2/SD^2, where β is the effect size of the allele and SD is the standard deviation) to assess the strength of the relationship between instrumental variables and the phenotype, where F > 10 indicates a strong correlation between instrumental variables and the phenotype (Von Hinke et al., 2011). All the aforementioned Mendelian Randomization-related statistical analyses were conducted using the TwoSampleMendelian Randomization package in R 4.3.2 software.

### Sequencing data acquisition and preprocessing

Using GENECARD, we retrieved genes associated with both AF and GC, selecting genes with correlation coefficients greater than 10 to form the relevant gene set. Subsequently, we downloaded the GSE251990, GSE62254 dataset from the GEO website, comprising single-cell data from 22 cases of normal gastric tissue, intestinal tissue, and GC, for subsequent analysis. It is important to emphasize that all data reanalyzed in this study were previously publicly available in prior reports.

### Construction and filtering of the interaction network of key genes

By intersecting the genes previously retrieved, we obtained a set of genes co-related with both AF and GC, serving as the key gene set. Subsequently, we uploaded these genes to the STRING online database (http://cn.string-db.org/) and set the confidence threshold to be greater than 0.4. We chose to hide disconnected nodes as the filtering criterion.

### Functional analysis of gene sets

To gain deeper insights into the functional significance of the identified key genes, gene ontology (GO) and Kyoto Encyclopedia of Genes and Genomes (KEGG) analyses were employed (Xu et al., 2023). Enrichment was determined using Fisher’s exact test, considering p < 0.05 as significant. The methodology for KEGG pathway enrichment analysis followed similar principles, aiding in comprehensively understanding the biological processes influenced by the AF-related gene set on GC. The “scMetabolism” R package utilizing the VISION method enabled quantification of metabolic activity at the single-cell resolution. This package encompasses 85 KEGG pathways, facilitating comprehensive analysis. Metabolic activity across different clusters was assessed using scMetabolism, and the results were visualized using the DotPlot.metabolism function (Jin et al., 2020).

### Preprocessing of single-cell RNA-seq data

The raw single-cell RNA sequencing (scRNA-seq) data were processed using the Seurat R package (version 5.0) to remove low-quality cells and visualize the data (Hao et al., 2023). Cells were filtered out if they exhibited characteristics such as having more than 200 genes per cell or having mitochondrial genes exceeding 20%. Subsequently, after identifying the top 2,000 highly variable genes (HGV), normalization and scaling of the scRNA-seq data were performed using Seurat. The harmony R package (version 0.1.1) was utilized to address batch effects among samples. Principal component analysis (PCA) was then conducted, and 20 principal components (PCs) were selected. Clusters were generated using two-dimensional Uniform Manifold Approximation and Projection (UMAP) visualization based on the selected PCs, followed by identification of cell clusters using the “FindClusters” function. Cell types were annotated based on well-established marker genes, and highly expressed genes within each cell cluster were identified using Seurat. UMAP plots were created using the “RunUMAP” function based on the top 30 principal components. Cell type annotations were performed using known cell type-specific markers and other cell markers from previous studies, and the proportions of cell types at each stage were calculated. Additionally, the R package AUCell was employed to score cell subpopulations and different samples based on the expression levels of the key gene set.

### Assessing cellular stemness using CytoTRACE

CytoTRACE introduces a novel framework for computing cellular differentiation potential (Gulati et al., 2020). This framework utilizes gene counts at the single-cell level to significantly enhance the assessment of cellular differentiation. Unlike most existing lineage trajectory analysis methods, CytoTRACE can predict relative states and differentiation directions, regardless of specific time scales or the presence of continuous developmental processes in the data. In this study, CytoTRACE was employed to compute the stemness score of fibroblast cells.

### Pseudotime trajectory analysis

We utilized Monocle two to establish potential developmental trajectories between cell subtypes (Qiu et al., 2017). To examine the developmental trajectory of fibroblast cells, we employed the Seurat v5.0 FindVariableFeatures function to select the top 2,000 highly variable genes from cell clusters. Subsequently, a principal tree was constructed using DDRTree to elucidate the progression of individual cells throughout the biological process and reconstruct their trajectories, while also calculating the key regulatory genes.

### Single-cell transcription factor regulatory network analysis

To identify key transcription factors (TFs) in different cell types, we performed cis-regulatory analysis using SCENIC (version 1.3.1) (Aibar et al., 2017; Suo et al., 2018). SCENIC is a tool based on co-expression and DNA motif analysis used to infer gene regulatory networks. Subsequently, we evaluated the network activity of each cell by calculating the area under the curve (AUC). In summary, we employed SCENIC to identify transcription factors, assembling them into modules (regulons), and analyzed them using RcisTarget with gene motif rankings: 500 bp upstream and 100 bp downstream of the transcription start site (TSS). Then, we used AUCell to score the activity of regulons in each cell in the dataset and visualized the results.

### Analysis of meta-programs expression

A meta-progam (MP) refers to a set of genes co-expressed in cells or tissues, which may participate in specific biological processes or phenotypes. These MPs may represent different biological processes or subtypes, aiding in the understanding of transcriptional heterogeneity at different stages of tumorigenesis (Gavish et al., 2023). We downloaded the gene list of MPs, scored each cell using AUCell, and visualized the results.

### Establishment of nomogram and ROC curve

This study utilized TCGA-STAD data to establish a Nomogram. The Nomogram was developed based on three parameters: age, TNM classification, pathological stage, and the expression of core genes. ROC curves were generated for the Nomogram.

### Mutation analysis

To analyze mutation data and clinical details, we utilized the “maftools” R package. The function “read.maf” was employed to import information from TCGA-STAD into the MAF file format. Subsequently, we utilized “plotmafSummary” to examine the mutation landscape of STAD patients in the TCGA dataset, visualizing the mutation status of pivotal genes.

### Statistical analysis

This study employed R for statistical assessment and data analysis. Kruskal–Wallis test was utilized for multi-group differential analysis, where p < 0.05 was considered statistically significant. Survival analysis was conducted using Kaplan-Meier method and Cox proportional hazards regression model. Differences in survival curves were assessed using the log-rank test. Multivariate Cox regression analysis was performed on the constructed core genes to determine their prognostic value (Rich et al., 2010). A p-value less than 0.05 was considered statistically significant. Hazard ratios (HR) and their 95% confidence intervals (CI) were computed to assess the risk associated with each gene in the signature.

## Results

### Two-sample mendelian randomization revealed the potential causal effect of AF on the risk of GC

**FIGURE 3:**
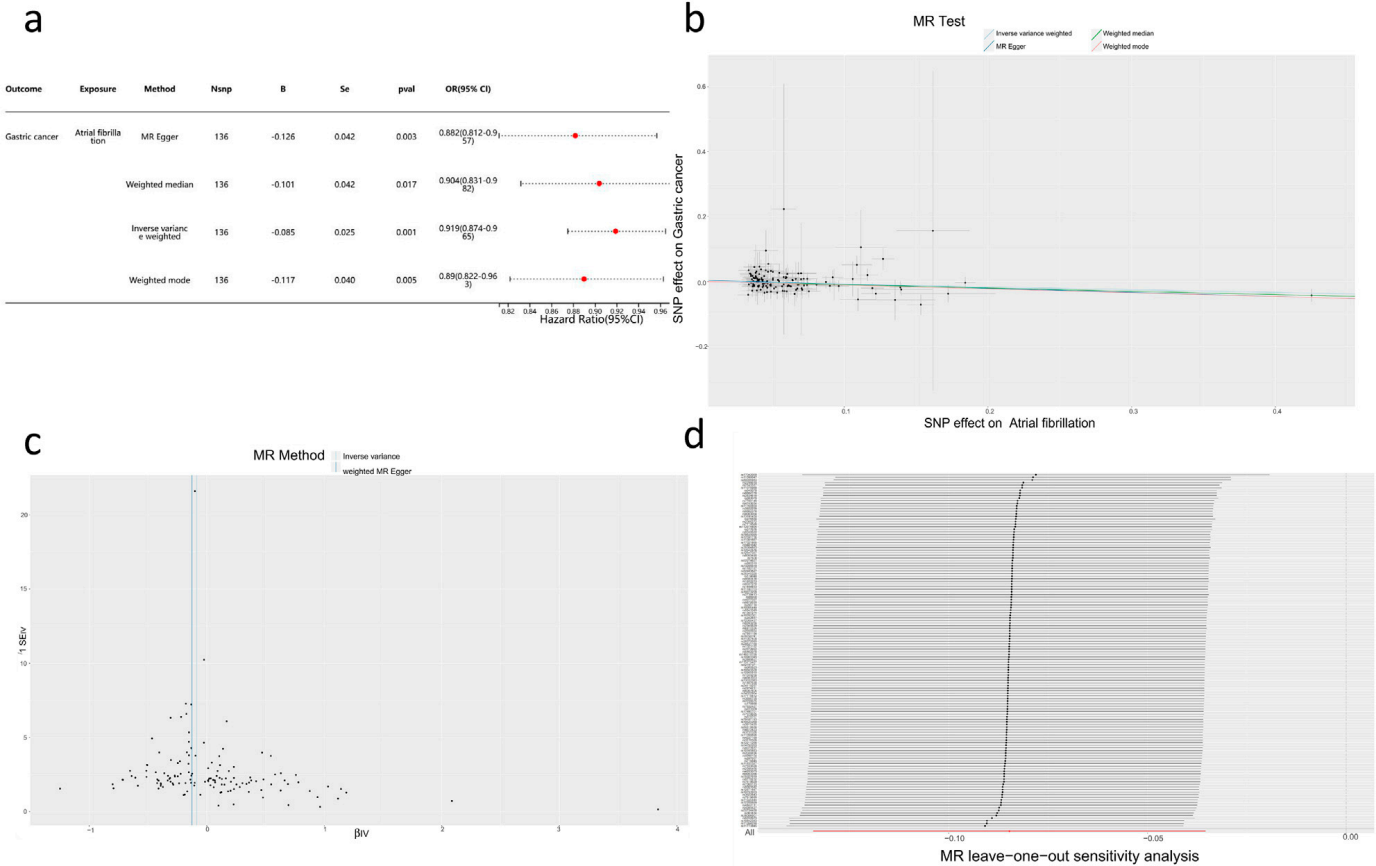
Mendelian Randomization Study Results **(A)** Forest plot of four assessment methods from the two-sample Mendelian Randomization study with AF as the exposure factor and GC as the outcome factor. **(B)** Scatter plot showing the causal effect of AF on the risk of GC. **(C)** Sensitivity analysis of the causal impact of AF on the risk of GC. **(D)** Funnel plot illustrating the causal effect of AF on the risk of GC.

Based on the methods described earlier, this study constructed a genetic instrument consisting of 136 SNPs to reveal the potential causal effect of AF on the risk of GC (Supplementary Table S1, S2). Through the inverse variance-weighted method, a negative correlation between AF and GC occurrence was observed (OR = 0.919, 95% CI = 0.88–0.96, p < 0.001). Consistent results of risk estimation (OR = 0.88–0.90) were observed across various methods, all of which were statistically significant (Figures 3A, B), and no evidence of pleiotropy or directional pleiotropy was found. Sensitivity analysis and funnel plot results supported the aforementioned findings (Figures 3C,D).

### Two-sample mendelian randomization reveals the relationship between AF and other risk factors for GC

Further research indicates that there is no causal relationship between AF and other risk factors for GC, such as chronic gastritis, *Helicobacter pylori* infection, and alcohol consumption (Supplementary Material S2). This suggests that the association between AF, determined by genetics, and the risk of GC is not influenced by these specific risk factors. This further supports the validity of the Mendelian Randomization study results.

### Construction of key genes involved in the impact of AF on GC and analysis of protein interaction networks

**FIGURE 4:**
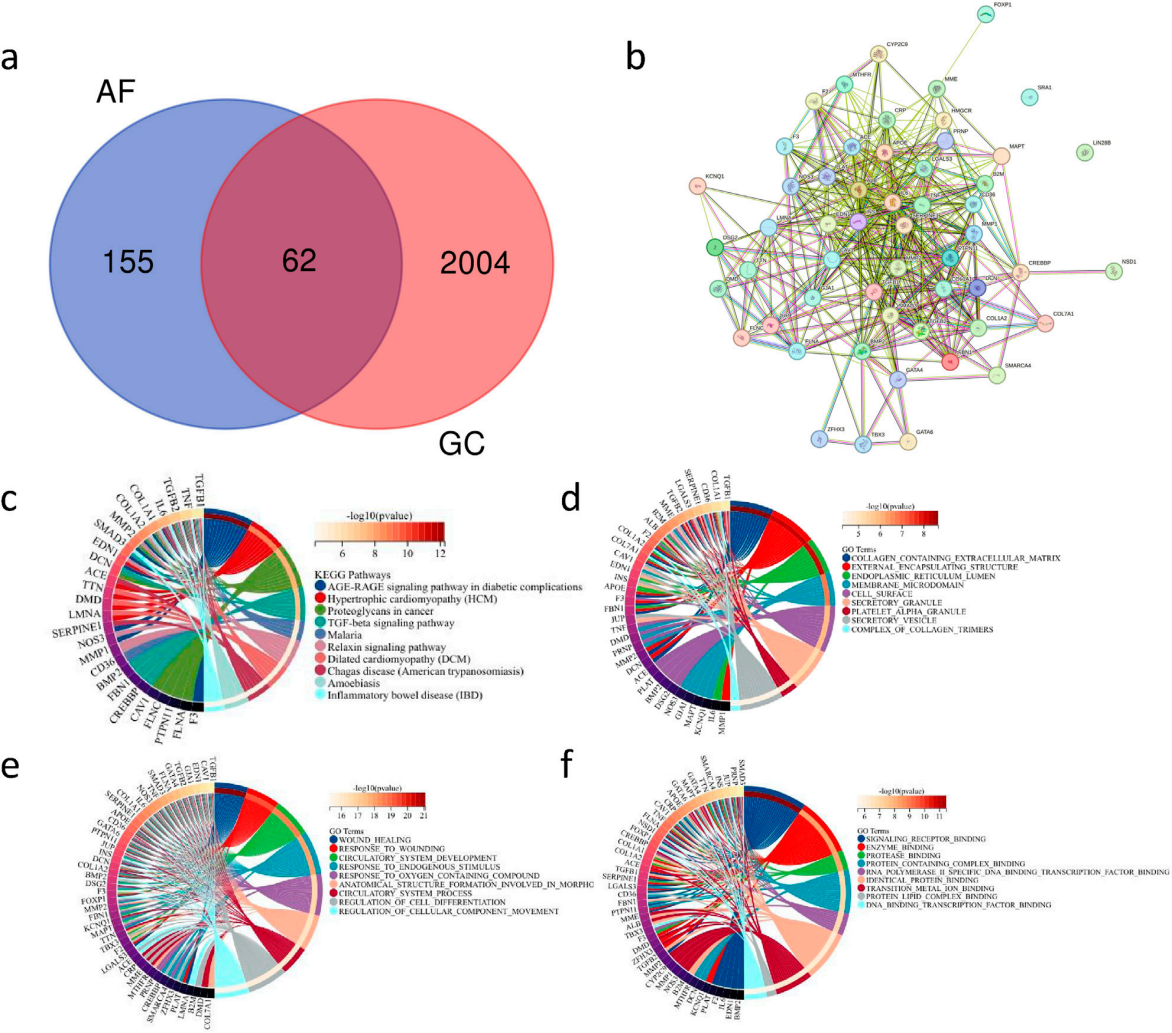
Key Gene Set of Co-associated Genes between AF and GC and Their PPI Network Construction with Functional Enrichment Analysis **(A)** Venn diagram showing the intersection of 217 AF-related genes and 2066 GC-related genes. **(B)** Protein-protein interaction (PPI) network diagram of the 62 key genes. **(C)** Circular plot depicting the enrichment results of the key gene set in the KEGG database (fdr <0.01, p < 0.05). **(D)** Circular plot displaying the enrichment results of the key gene set in the GO:CC database (fdr <0.01, p < 0.05). **(E)** Circular plot illustrating the enrichment results of the key gene set in the GO:BP database (fdr <0.01, p < 0.05). **(F)** Circular plot demonstrating the enrichment results of the key gene set in the GO:MF database (fdr <0.01, p < 0.05).

Through GeneCard, we obtained a total of 217 genes associated with AF and 2066 genes related to GC. By intersecting the genes associated with AF and those associated with GC, we identified 62 genes as the common correlated genes (Figure 4A), serving as the key gene set. Further, utilizing the STRING database, we constructed a protein-protein interaction network for the key gene set (Figure 4B).

### Enrichment analysis of potential mechanisms of key gene set

Enrichment analysis using the KEGG database (Figure 4C) and GO databases (Figures 4D–F) investigated the potential mechanisms of the key gene set. The results of the enrichment analysis revealed associations of the key gene set with various pathways related to cardiovascular diseases, inflammatory gastrointestinal diseases, and tumorigenesis. These findings suggest that the key gene set may be involved in the pathogenesis of multiple diseases, tumor mutations, and carcinogenic pathways.

### Analysis of GC cell subtypes and identification of key subtypes

**FIGURE 5:**
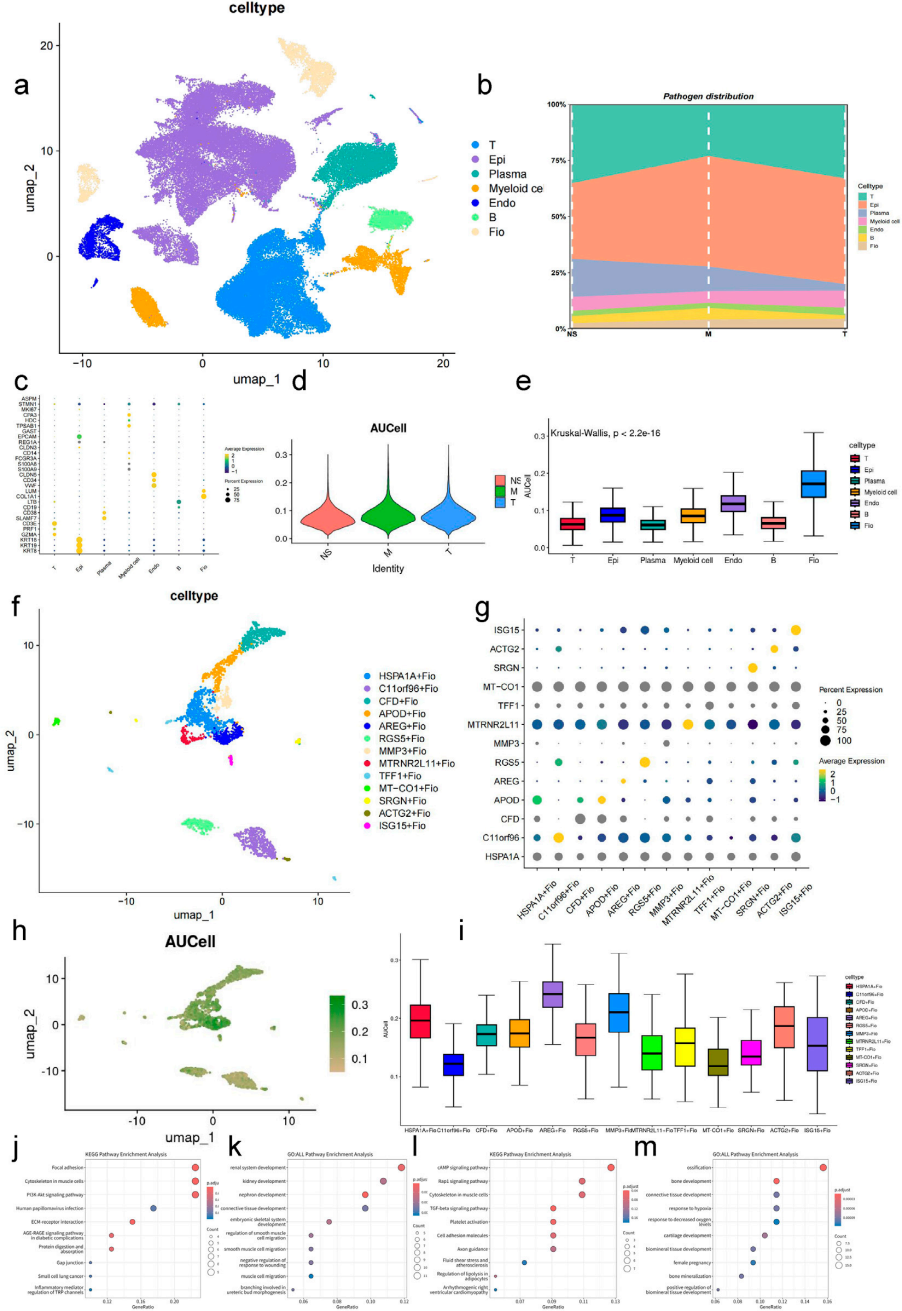
Expression Analysis of Key Gene Set in GC-Associated Single-Cell Data **(A)** UMAP plot of integrated single-cell data. **(B)** Bar plot showing the cellular composition across three stages in the single-cell sequencing data. **(C)** Bubble plot of cluster markers. **(D)** Violin plot displaying AUCell scores of the key gene set across three stages of samples. **(E)** Box plot showing AUCell scores of the key gene set across different cell types (Kruskal–Wallis, p < 0.001). **(F)** UMAP plot of fibroblast clustering. **(G)** Bubble plot of top markers for fibroblast subgroups. **(H)** UMAP plot displaying AUCell scores of the key gene set in fibroblasts. **(I)** Box plot showing AUCell scores of the key gene set in fibroblast subgroups (Kruskal–Wallis, p < 0.001). **(J)** Bubble plot showing the top 10 enriched pathways in the KEGG database for marker genes of the HSPA1A + Fio subgroup (fdr <0.01, p < 0.05). **(K)** Bubble plot showing the top 10 enriched pathways in the GO database for marker genes of the HSPA1A + Fio subgroup (fdr <0.01, p < 0.05). **(L)** Bubble plot showing the top 10 enriched pathways in the KEGG database for marker genes of the AREG + Fio subgroup (fdr <0.01, p < 0.05). **(M)** Bubble plot showing the top 10 enriched pathways in the GO database for marker genes of the AREG + Fio subgroup (fdr <0.01, p < 0.05).

By analyzing single-cell sequencing data from normal gastric tissue, intestinalized tissue, and GC obtained from the GEO database, we identified seven major subgroups: epithelial cells (Epi), endothelial cells (Endo), B cells (B), myeloid cells (Myeloid cell), T cells (T), fibroblasts (Fio), and plasma cells (Plasma cell) (Figure 5A), and analyzed the composition of cells at three stages (Figure 5B). The markers used for identification were derived from previous studies, as shown in Figure 5C.

Using the previously identified key gene set, we performed AUC scoring for cell subgroups and pathological stages (Figures 5D, E). The results showed significant differences in AUC scores of cells at different developmental stages and disease states (p < 0.01). Specifically, fibroblasts had the highest AUC score, thus we selected fibroblasts for subsequent analysis.

After subtyping fibroblasts, we identified differential genes using the COSG method and excluded genes with FDR values greater than 0.05. Subsequently, we named cell subgroups based on the gene with the maximum LOG2FC, resulting in the identification of 13 fibroblast subgroups (Figures 5F, G). Further analysis revealed that the scores of HSPA1A + Fio, ARGE + Fio, and MMP3+Fio subgroups within fibroblasts were higher than other subgroups, indicating that these may be key subgroups for the effect of AF-related genes on GC (Figures 5H, I). Enrichment analysis of the top 100 genes calculated by the COSG method for ARGE + Fio and HSPA1A + Fio subgroups using AUCell revealed significant associations with cell adhesion, inflammation regulation, receptor activity, cell development, and blood circulation pathways, similar to our previous key gene set enrichment results (Figures 5J–M).

### Fibroblast differentiation, developmental trajectory analysis, and transcription factor regulation revealed its potential role in GC development

**FIGURE 6:**
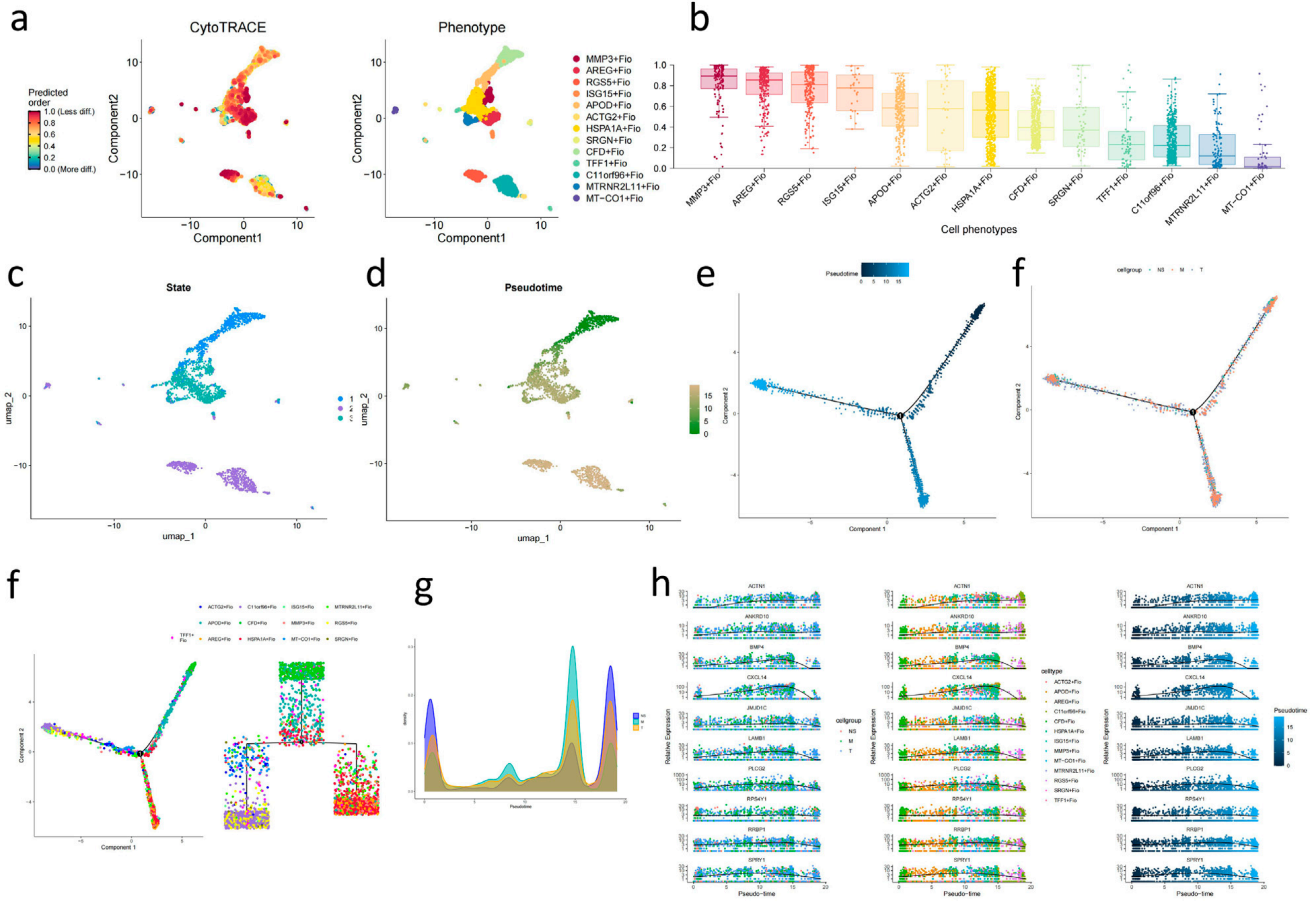
Analysis of Stemness and Developmental Trajectory of Fibroblast Subgroups **(A)** UMAP plot of fibroblast subpopulations’ stemness assessed by CytoTrace. **(B)** Boxplot showing the assessment of fibroblast subpopulations’ stemness based on CytoTrace. **(C)** UMAP plot of fibroblast subpopulations’ different states evaluated by Monocle2. **(D)** UMAP plot depicting the developmental time sequence of fibroblast subpopulations based on Monocle2. **(E)** Developmental trajectory plot of fibroblasts (based on inferred developmental time). **(F)** Developmental trajectory plot of fibroblasts (based on sample pathological stages). **(G)** Phylogenetic tree of fibroblast lineage. **(H)** Mountain plot illustrating the distribution of pathological stages during fibroblast development. **(I)** Line chart displaying genes influencing fibroblast development.

**FIGURE 7:**
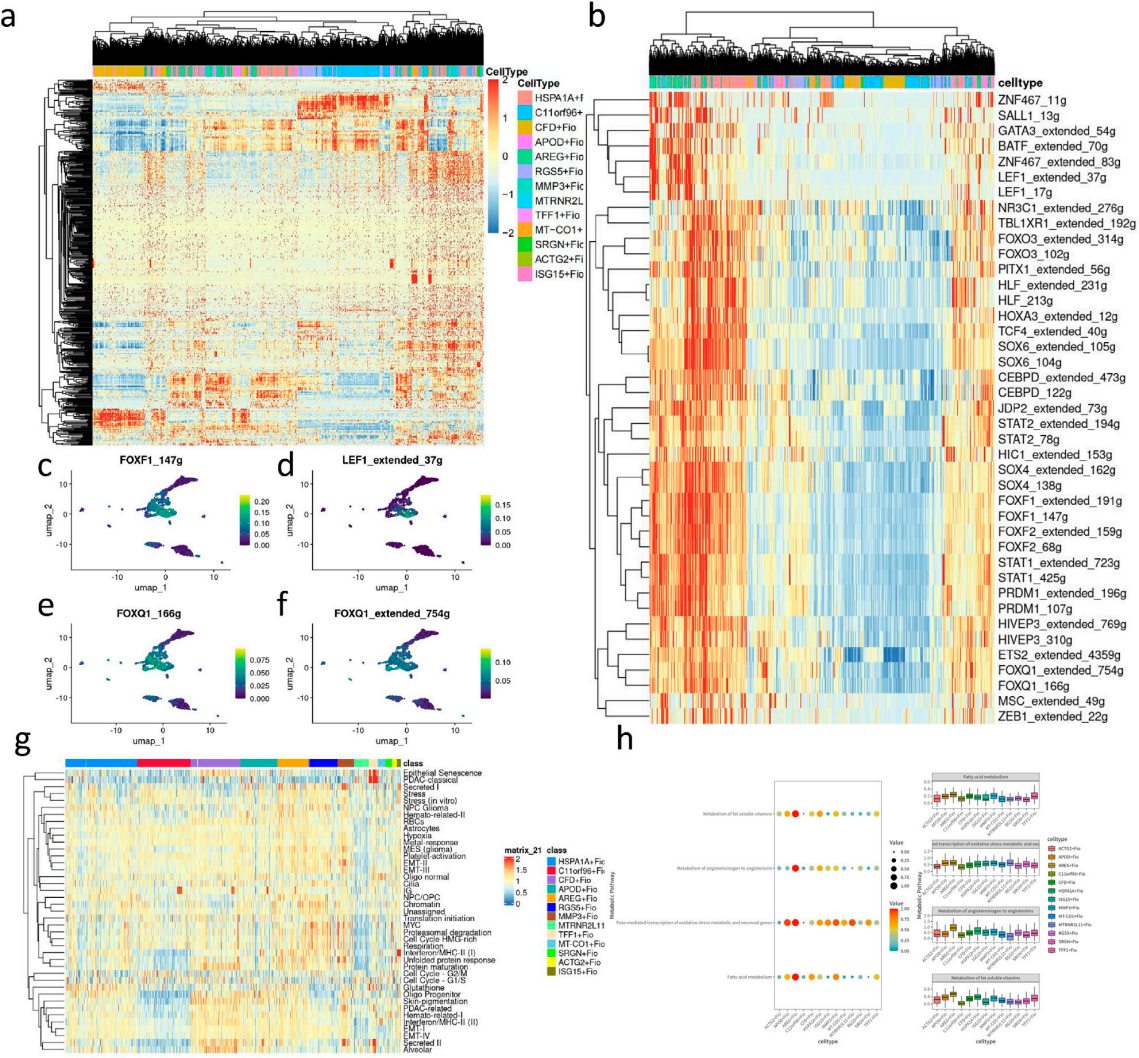
Transcription factor, cell metaprogram, and metabolic pathway analysis of fibroblast subpopulations. **(A)** Heatmap of transcription factor analysis in fibroblasts. **(B)** Heatmap of upregulated transcription factors in key subpopulations. **(C)** UMAP plot depicting the expression level of FOXF1_147 g in fibroblasts. **(D)** UMAP plot illustrating the expression level of LEF1 extended 37 g in fibroblasts. **(E)** UMAP plot showing the expression level of FOXQ1_166 g in fibroblasts. **(F)** UMAP plot displaying the expression level of FOXQ1 extended 754 g in fibroblasts. **(H)** Heatmap of cancer-associated metaprogram scores in fibroblast subpopulations. **(I)** Enriched metabolic pathways in key upregulated subpopulations based on SCmetabolism analysis (Kruskal–Wallis, p < 0.001).

Through CytoTRACE assessment of fibroblast subgroups (Figures 6A, B), the HSPA1A + Fio subgroup was found to belong to more mature and less stem-like fibroblasts, while the ARGE + Fio and MMP3+Fio subgroups exhibited lower differentiation and stronger stem-like characteristics. Subsequently, we conducted pseudotime analysis using MONOCLE2, revealing the developmental trajectory of fibroblasts (Figures 6C–H). We observed significant expression changes in genes such as ACTN1, ANKRD10, and BMP4 during the developmental process (Figure 6I). Subsequent transcription factor analysis of fibroblasts (Figures 7A, B) revealed significant upregulation of transcription factors associated with GC development, including FOXF1, LEF1, and FOXQ1, in key subgroups (Figures 7C–F).

### Differential cancer-associated metaprogram and metabolic regulatory features in fibroblast subpopulations

We performed cancer-associated metaprogram expression analysis in fibroblasts, using AUCell to score each cell based on previously reported cancer-associated metaprograms. Among the key subpopulations, processes such as Secreted I and EMT-II, which are associated with tumor progression, were upregulated (Figure 7G). scMetabolism was utilized to measure single-cell metabolic activities, revealing differences in metabolic pathways among 13 clusters. Particularly, the ARGE + Fio cluster exhibited higher activity in pathways such as fatty acid metabolism and fat-soluble vitamin metabolism, showing significant metabolic differences compared to other fibroblast subpopulations (Figure 7H).

### Prognostic analysis and mutation validation of key gene sets based on the TCGA-STAD cohort

**FIGURE 8:**
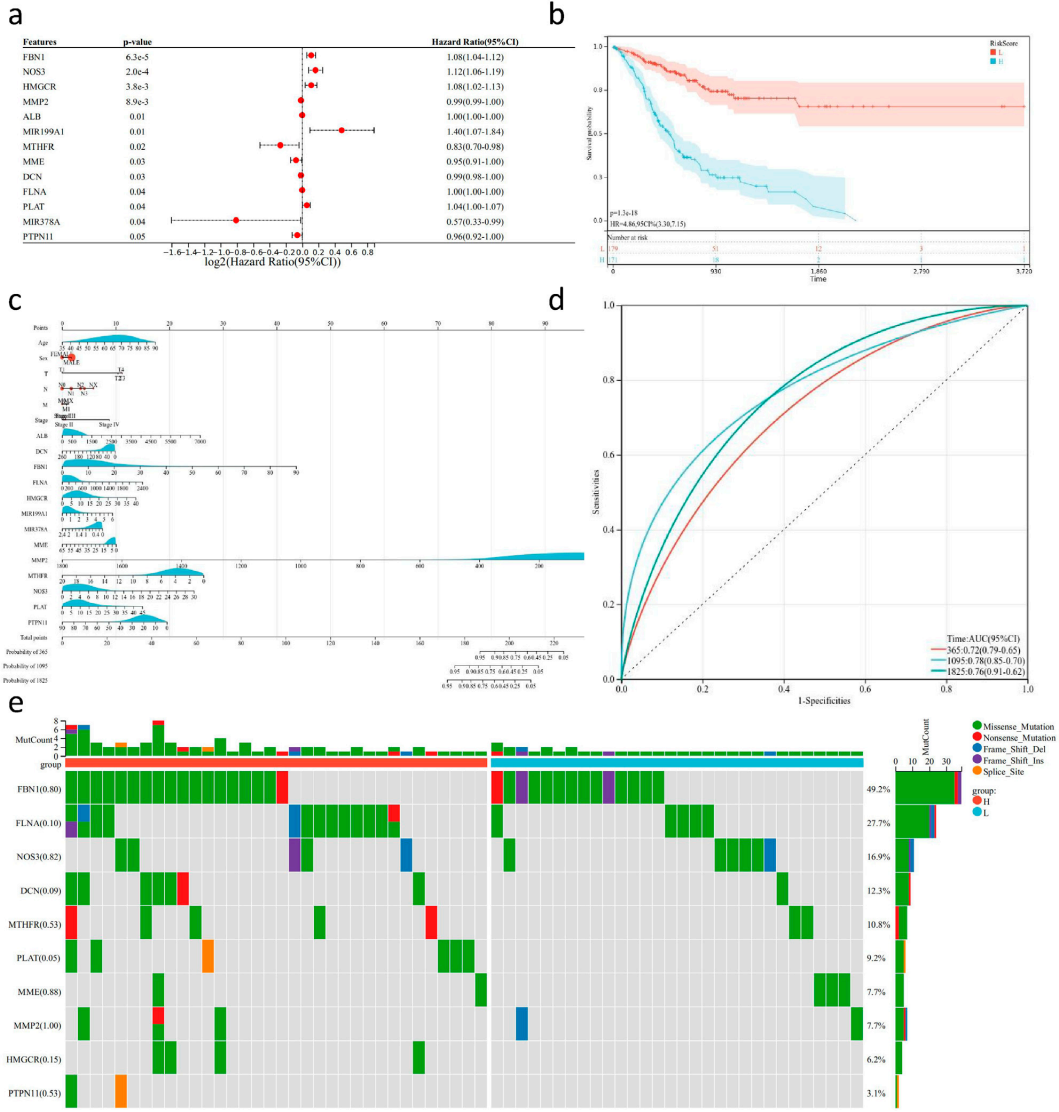
Survival analysis and mutation detection of key genes in GC transcriptome sequencing data. **(A)** Forest plot of 13 risk genes obtained from multi-factor Cox analysis based on TCGA-STAD transcriptome sequencing data and follow-up results (P < 0.05). **(B)** KM survival curve based on the risk genes using TCGA-STAD transcriptome sequencing data and follow-up results. **(C)** Nomogram generated based on the risk genes, TCGA-STAD transcriptome sequencing data, and follow-up results. **(D)** ROC curve evaluating the predictive ability of the nomogram. **(E)** Waterfall plot depicting the mutation status of risk genes in TCGA-STAD WES sequencing data.

In this study, we utilized the R package “survival” to integrate survival time, survival status, and data from key gene sets. Cox proportional hazards regression analysis was performed to evaluate the prognostic significance of these features in a cohort of 350 samples from TCGA-STAD (Figure 8A). The overall prognostic difference was found to be significant (logtest = 3.2e-05, sctest = 2.1e-05, waldtest = 0.00021), with a C-index of 0.719. Additionally, we identified 13 distant genes with prognostic significance. Using the R package “maxstat” (Maximally selected rank statistics with several p-value approximations version: 0.7–25), we calculated the optimal cutoff value for RiskScore. The minimum sample size in each group was set to be greater than 25%, and the maximum sample size was set to be less than 75%. The optimal cutoff value was determined to be −1.44. Based on this, patients were divided into high and low-risk groups. Further analysis using the “survfit” function in the R package “survival” revealed significant prognostic differences the groups using the log-rank test (p = 1.3e-) (Figure 8B). Based on these results we constructed a nomogram to predict the prognosis of GC patients. The nomogram included multiple predictive variables such as age, TMN staging, pathological stage, and key gene sets, and demonstrated good predictive performance (Figure 8C). ROC results showed that the classifier model on the key gene set exhibited good performance in distinguishing between and negative samples, areas under the curve of 0.72, 0.78, and 0.76 for predicting one, and 5-year survival rates, respectively (Figure 8D). To further verify the robustness of the model we constructed, we used the GSE62254 gastric cancer database for further external validation. We included the previous factors in the same way, with the additional factor of gender to calculate the accuracy of their predictions. We found that the model had good predictive power, and the predicted survival of the high-risk group was significantly lower than that of the low-risk group (P < 0.05,HR = 4.17, Supplementary Figure S5). At the same time, we drew the ROC curve to verify the prediction efficiency of the model, and found that the ROC values at the 1-year, 3-year, and 5-year nodes were 0.81, 0.78, and 0.75, respectively, indicating better prediction ability and proving the reliability of predicting the survival of gastric cancer patients based on the previous risk genes (Supplementary Figure S6). Additionally, we validated the mutation status of the key set using exome sequencing data from the TCGA-STAD cohort (Figure 8E). A total of 332 samples were evaluated for mutations, and the plotted samples included 64 (19.3%). We used chi-square test to assess the differences in mutation frequency for each gene between sample groups. Among all genes, FBN had the highest mutation frequency, reaching 49.2%, with predominantly missense mutations. Furthermore, we observed a significantly higher mutation rate of PLAT in the high-risk group compared to the low-risk group.

## Discussion

In this study, we conducted a two-sample Mendelian Randomization analysis to thoroughly evaluate whether AF has a causal effect on GC incidence. Our results supported the causal effect of AF genetic predisposition on the risk of GC, showing an inverse correlation between them. Subsequently, by analyzing the causal relationship between AF and GC risk factors, we found that the association between AF and GC is not influenced by these specific GC risk factors, further supporting the validity of previous research findings. We then constructed an AF-GC-related gene set and performed various analyses using GC bulk and single-cell sequencing data. The analysis results showed that this gene set is mainly associated with fibroblasts and that there are specific fibroblast subpopulations in GC with higher AF-GC-related gene set scores, suggesting their potential widespread impact on GC. Subsequently, using GC transcriptome sequencing data, we confirmed the correlation of this gene set with poor prognosis in GC patients and developed a predictive model that demonstrated good performance. Our study revealed the causal relationship between AF and GC incidence, as well as the potential mechanisms by which AF-related genes influence GC progression.

In a study involving 25, 964, 447 participants, it was found that 61.44% of cancer patients had complications of AF (Chen et al., 2023). Additionally, other studies have shown that AF is associated with an average 1.4-fold increase in cancer incidence (Joseph et al., 2020). Subsequent research has suggested that long-term chronic inflammation-induced immune activation may be a potential cause of AF and angina related to gastrointestinal diseases (Chan et al., 2014; Shi et al., 2020). These findings suggest the need to explore the potential causal relationship between GC and AF. Furthermore, contemporary studies also indicate that cardiovascular diseases may interact with gastrointestinal diseases through mechanisms such as inflammation, metabolism, immunity, and circulation (Budzyński et al., 2014), although the specific pathways are not yet clear, and the potential impact mechanisms of AF-related genes on GC remain to be elucidated. To understand the potential impact of AF on GC, we first evaluated the functional relevance of shared genes between AF and GC, and found enrichment of the TGF pathway and Relaxin signaling pathway. The TGF pathway is a classical cancer-related signaling pathway (Massagué, 2012), and its activation has been reported to contribute to the killing of some precancerous cells, while promoting cancer invasion and metastasis. The activation of the TGF pathway may be a key pathway through which AF-related genes influence GC. The Relaxin signaling pathway mainly includes anti-fibrosis, vasodilation, angiogenesis, anti-inflammatory, anti-apoptotic, and organ protective effects, and is considered a potential therapeutic target for GC (Sheng et al., 2018; Wang et al., 2023). We then located the relevant gene action through single-cell data, which indicated that it mainly acts on fibroblasts. Fibroblasts can secrete specific cytokines and extracellular matrix components, affecting the malignant biological behavior of tumor cells such as proliferation, metastasis, and drug resistance (Chen et al., 2021). Therefore, we further investigated fibroblasts. We identified specific subpopulations enriched with TNF signaling pathway, cell development, inflammation-mediated processes, which are consistent with our previous findings. We evaluated the stemness of fibroblast subpopulations based on single-cell data and identified differentiation trajectories of fibroblasts. We found that subpopulations with high AUCell scores (AREG + Fio, HSPA1A + Fio, MMP3+Fio) exhibited distinct differentiation trajectories compared to other cells. BMP4 and CXCL14 were identified as key genes in the differentiation process, showing upregulation in the high AUCell score subpopulations. It has been reported that high expression of BMP4 in fibroblasts is closely related to GC formation and gastric intestinalization (Tsubosaka et al., 2023). CXCL14 is a homeostatic chemokine and its role in tumors is bidirectional. It is associated with overall survival in colorectal cancer, breast cancer, endometrial cancer, epithelial cancer, and head and neck cancer (Giacobbi et al., 2024). It can inhibit tumor growth but has a tumor-promoting effect in glioblastoma, non-small cell lung cancer, and microsatellite-stable colorectal tumors. However, its role in GC remains to be studied. We further identified upregulated transcription factors in these specific fibroblast subpopulations through transcription factor analysis, such as FOXF1 (a tumor suppressor transcription factor) (Bian et al., 2024) and FOXQ1 (a tumor-promoting transcription factor) (Wu et al., 2023). These changes may ultimately affect the proliferation and metastasis of GC. FOXF1 and CXCL14 may be the reasons for the negative correlation between AF and GC risk. Based on the expression of cancer-associated metaprograms, the expression of Secreted l was significantly upregulated in specific subpopulations, and it is associated with blood circulation and tumor immunity. We further assessed the metabolic pathways of fibroblast subpopulations using the SCmetabolism method and found that the angiotensin and lipid metabolism processes were significantly upregulated in the AREG + Fio subpopulation, indicating that the key gene set may affect GC by regulating the differentiation of fibroblasts (Din et al., 2024). Finally, we used sequencing data and survival information from TCGA to evaluate the genes associated with GC risk in the gene set, and identified 13 risk genes: FBN1, NOS3, HMGCR, MMP2, ALB, MIR199A1, MTHFR, MME, DCN, FLNA, PLAT, MIR378A, and PTPN11. Based on these genes, we performed K-M survival curve analysis, nomogram construction, ROC curve analysis, and mutation analysis, and found that they had good predictive ability for GC prognosis, further confirming the impact of this gene set on GC.

Although we have made some important findings, we also need to acknowledge the limitations of this study. Firstly, most of the GWAS datasets are derived from European populations, so it is still important to validate the study findings across different racial and ethnic groups. Secondly, future research can consider expanding the study to different subtypes of GC to gain a more comprehensive understanding of the association between AF and different types of GC. Finally, this study mainly focuses on reporting the potential association and molecular mechanism between atrial fibrillation and gastric cancer. Further *in vitro* and *in vivo* studies and clinical sample collection are needed in the follow-up study to further explore the relationship between the two. Moreover, comprehensive longitudinal examination through real-world studies to assess the impact of AF diagnosis and treatment on subsequent gastric cancer risk is also necessary.

## Conclusion

Overall, the results of our study supplied genetic evidence suggesting that the genetic liability to AF reduces the risk of GC, and identify potential pathways by which some AF related genes may influence gastric cancer. This study offers important references and insights for future research and clinical practice. We look forward to further exploring the role of AF-related genes in the pathogenesis of GC, to promote a deeper understanding of the association between these two diseases and future directions for treatment.

## Data Availability

The datasets presented in this study can be found in online repositories. The names of the repository/repositories and accession number(s) can be found in the article/Supplementary Material.

## Abbreviations

AF, Atrial fibrillation; GC, Gastric cancer; OS, Overall survival; GO, Gene ontology; KEGG, Kyoto Encyclopedia of Genes and Genomes; LOG2FC, Log2 Fold Change; ROC, Receiver operating characteristic.
