# Supplementary material for: Mendelian randomization and multiomics comprehensively reveal the causal relationship and potential mechanism between atrial fibrillation and gastric cancer
Source: Front Genet. 2025 Feb 3;16:1446661. doi: 10.3389/fgene.2025.1446661 (PMC11830663; doi:10.3389/fgene.2025.1446661)

**Supplementary materials**

**Supplementary Table 1.** The summary information for instrumental variables of Atrial fibrillation

| **Sort** | **Phenotype** | **SNP** | **effect_allele** | **other_allele** | **beta** | **se** | **pval** |
| --- | --- | --- | --- | --- | --- | --- | --- |
| 1 | Atrial fibrillation | rs74832855 | G | A | 0.1425 | 0.0199 | 7.46E-13 |
| 2 | Atrial fibrillation | rs4484922 | C | G | -0.063 | 0.0078 | 4.57E-16 |
| 3 | Atrial fibrillation | rs2885697 | T | G | -0.0391 | 0.0075 | 1.77E-07 |
| 4 | Atrial fibrillation | rs61750827 | T | C | 0.0769 | 0.0165 | 3.11E-06 |
| 5 | Atrial fibrillation | rs11264280 | T | C | 0.127 | 0.0078 | 4.60E-59 |
| 6 | Atrial fibrillation | rs2813865 | G | A | 0.0482 | 0.0089 | 6.97E-08 |
| 7 | Atrial fibrillation | rs10753933 | G | T | -0.0743 | 0.0072 | 5.83E-25 |
| 8 | Atrial fibrillation | rs146518726 | A | G | 0.1617 | 0.0254 | 2.05E-10 |
| 9 | Atrial fibrillation | rs79187193 | A | G | -0.1116 | 0.0182 | 8.07E-10 |
| 10 | Atrial fibrillation | rs7549338 | G | C | -0.0454 | 0.0071 | 1.71E-10 |
| 11 | Atrial fibrillation | rs72700114 | C | G | 0.2026 | 0.0139 | 7.32E-48 |
| 12 | Atrial fibrillation | rs577676 | T | C | -0.0962 | 0.0072 | 4.38E-41 |
| 13 | Atrial fibrillation | rs6665642 | T | C | -0.0549 | 0.012 | 4.72E-06 |
| 14 | Atrial fibrillation | rs880315 | C | T | 0.0437 | 0.0075 | 5.04E-09 |
| 15 | Atrial fibrillation | rs17021865 | C | T | 0.1354 | 0.0281 | 1.47E-06 |
| 16 | Atrial fibrillation | rs2372992 | G | A | -0.0396 | 0.0077 | 3.01E-07 |
| 17 | Atrial fibrillation | rs35927619 | G | T | -0.0375 | 0.0076 | 9.07E-07 |
| 18 | Atrial fibrillation | rs35504893 | T | C | 0.09 | 0.0087 | 6.89E-25 |
| 19 | Atrial fibrillation | rs10165883 | T | C | -0.0642 | 0.0072 | 5.83E-19 |
| 20 | Atrial fibrillation | rs2540949 | T | A | -0.0752 | 0.0073 | 8.17E-25 |
| 21 | Atrial fibrillation | rs35215597 | G | A | -0.0764 | 0.0086 | 5.91E-19 |
| 22 | Atrial fibrillation | rs4663039 | A | G | 0.0611 | 0.0128 | 1.69E-06 |
| 23 | Atrial fibrillation | rs6546620 | C | T | 0.0708 | 0.0093 | 2.96E-14 |
| 24 | Atrial fibrillation | rs34118297 | T | C | 0.0464 | 0.0098 | 2.25E-06 |
| 25 | Atrial fibrillation | rs295114 | T | C | -0.0676 | 0.0073 | 1.76E-20 |
| 26 | Atrial fibrillation | rs62197371 | A | C | -0.0402 | 0.0079 | 3.65E-07 |
| 27 | Atrial fibrillation | rs143851124 | G | A | -0.0443 | 0.0093 | 1.72E-06 |
| 28 | Atrial fibrillation | rs2044456 | G | A | 0.037 | 0.0077 | 1.64E-06 |
| 29 | Atrial fibrillation | rs12992412 | T | A | 0.0406 | 0.0073 | 2.30E-08 |
| 30 | Atrial fibrillation | rs4672423 | T | C | -0.0426 | 0.0073 | 5.85E-09 |
| 31 | Atrial fibrillation | rs72926475 | A | G | -0.0708 | 0.0113 | 3.49E-10 |
| 32 | Atrial fibrillation | rs73206619 | T | G | 0.0407 | 0.0083 | 8.43E-07 |
| 33 | Atrial fibrillation | rs13066567 | A | G | 0.1056 | 0.0231 | 4.66E-06 |
| 34 | Atrial fibrillation | rs9872035 | T | C | -0.0374 | 0.0071 | 1.63E-07 |
| 35 | Atrial fibrillation | rs7632427 | C | T | -0.0425 | 0.0074 | 1.10E-08 |
| 36 | Atrial fibrillation | rs4855075 | T | C | 0.0604 | 0.0103 | 4.00E-09 |
| 37 | Atrial fibrillation | rs73032363 | G | A | -0.0432 | 0.0078 | 3.59E-08 |
| 38 | Atrial fibrillation | rs3856795 | A | G | 0.04 | 0.0076 | 1.36E-07 |
| 39 | Atrial fibrillation | rs3922843 | G | A | -0.0472 | 0.0082 | 8.99E-09 |
| 40 | Atrial fibrillation | rs2306272 | C | T | 0.0512 | 0.0078 | 4.54E-11 |
| 41 | Atrial fibrillation | rs17490701 | A | G | -0.07 | 0.0107 | 5.43E-11 |
| 42 | Atrial fibrillation | rs7618629 | G | A | -0.0384 | 0.0082 | 3.03E-06 |
| 43 | Atrial fibrillation | rs62274627 | A | G | 0.0363 | 0.0076 | 1.78E-06 |
| 44 | Atrial fibrillation | rs9862378 | T | G | 0.0332 | 0.0072 | 4.25E-06 |
| 45 | Atrial fibrillation | rs6810325 | C | T | 0.0747 | 0.0076 | 5.24E-23 |
| 46 | Atrial fibrillation | rs6790396 | G | C | 0.0636 | 0.0073 | 4.13E-18 |
| 47 | Atrial fibrillation | rs6778232 | C | T | -0.0409 | 0.0082 | 6.29E-07 |
| 48 | Atrial fibrillation | rs10213171 | G | C | 0.1041 | 0.0139 | 6.09E-14 |
| 49 | Atrial fibrillation | rs79835095 | T | C | 0.0445 | 0.0086 | 2.03E-07 |
| 50 | Atrial fibrillation | rs223369 | A | G | 0.041 | 0.0082 | 6.28E-07 |
| 51 | Atrial fibrillation | rs6841049 | G | T | -0.0366 | 0.0073 | 4.88E-07 |
| 52 | Atrial fibrillation | rs11935444 | C | T | -0.0335 | 0.0072 | 3.14E-06 |
| 53 | Atrial fibrillation | rs10520260 | G | A | -0.0539 | 0.0079 | 8.98E-12 |
| 54 | Atrial fibrillation | rs3822259 | T | G | 0.0463 | 0.0077 | 1.93E-09 |
| 55 | Atrial fibrillation | rs6838973 | T | C | -0.1842 | 0.0072 | 1.35E-142 |
| 56 | Atrial fibrillation | rs976568 | T | G | -0.139 | 0.0075 | 4.54E-76 |
| 57 | Atrial fibrillation | rs11099696 | T | C | -0.038 | 0.0072 | 1.37E-07 |
| 58 | Atrial fibrillation | rs12647387 | G | A | -0.0674 | 0.0135 | 6.14E-07 |
| 59 | Atrial fibrillation | rs297007 | A | C | -0.0387 | 0.0082 | 2.35E-06 |
| 60 | Atrial fibrillation | rs17042059 | A | G | 0.4252 | 0.0097 | 1.00E-200 |
| 61 | Atrial fibrillation | rs34750263 | T | C | 0.0873 | 0.0076 | 2.89E-30 |
| 62 | Atrial fibrillation | rs174048 | C | T | 0.0665 | 0.0098 | 1.05E-11 |
| 63 | Atrial fibrillation | rs78758741 | C | T | 0.0612 | 0.0122 | 4.79E-07 |
| 64 | Atrial fibrillation | rs7719528 | T | C | -0.036 | 0.0072 | 6.67E-07 |
| 65 | Atrial fibrillation | rs6882776 | A | G | -0.06 | 0.0079 | 3.17E-14 |
| 66 | Atrial fibrillation | rs115219487 | C | T | 0.0663 | 0.0125 | 1.28E-07 |
| 67 | Atrial fibrillation | rs716845 | A | G | 0.0594 | 0.008 | 1.16E-13 |
| 68 | Atrial fibrillation | rs17118812 | C | T | 0.0404 | 0.0078 | 2.41E-07 |
| 69 | Atrial fibrillation | rs12208899 | A | G | 0.049 | 0.0087 | 1.95E-08 |
| 70 | Atrial fibrillation | rs9481842 | G | T | 0.0655 | 0.0081 | 8.12E-16 |
| 71 | Atrial fibrillation | rs4896104 | T | C | -0.0351 | 0.0074 | 1.79E-06 |
| 72 | Atrial fibrillation | rs73366713 | A | G | -0.1052 | 0.0112 | 5.80E-21 |
| 73 | Atrial fibrillation | rs12211255 | A | C | 0.0593 | 0.0115 | 2.48E-07 |
| 74 | Atrial fibrillation | rs13191450 | C | A | -0.0704 | 0.0075 | 8.92E-21 |
| 75 | Atrial fibrillation | rs117984853 | T | G | 0.1132 | 0.0136 | 8.38E-17 |
| 76 | Atrial fibrillation | rs34969716 | A | G | 0.0875 | 0.0084 | 2.91E-25 |
| 77 | Atrial fibrillation | rs6907805 | T | G | -0.0405 | 0.0071 | 1.10E-08 |
| 78 | Atrial fibrillation | rs112974895 | C | A | -0.0584 | 0.0116 | 4.98E-07 |
| 79 | Atrial fibrillation | rs3176326 | A | G | -0.0599 | 0.0092 | 7.95E-11 |
| 80 | Atrial fibrillation | rs179968 | T | G | -0.0401 | 0.0082 | 1.13E-06 |
| 81 | Atrial fibrillation | rs1307274 | G | T | -0.0741 | 0.0135 | 3.85E-08 |
| 82 | Atrial fibrillation | rs62483627 | A | G | 0.0489 | 0.0084 | 5.17E-09 |
| 83 | Atrial fibrillation | rs55985730 | G | T | 0.0957 | 0.017 | 1.81E-08 |
| 84 | Atrial fibrillation | rs11773845 | A | C | 0.1162 | 0.0072 | 4.61E-58 |
| 85 | Atrial fibrillation | rs2949829 | T | C | -0.0352 | 0.0073 | 1.51E-06 |
| 86 | Atrial fibrillation | rs11768850 | T | C | 0.0392 | 0.0072 | 4.96E-08 |
| 87 | Atrial fibrillation | rs1182197 | C | A | -0.0373 | 0.0073 | 3.76E-07 |
| 88 | Atrial fibrillation | rs55734480 | A | G | 0.0504 | 0.0082 | 7.34E-10 |
| 89 | Atrial fibrillation | rs113455614 | G | C | 0.0694 | 0.0142 | 1.05E-06 |
| 90 | Atrial fibrillation | rs7789146 | A | G | -0.0571 | 0.0092 | 6.51E-10 |
| 91 | Atrial fibrillation | rs3731326 | G | A | -0.0455 | 0.0084 | 6.88E-08 |
| 92 | Atrial fibrillation | rs74910854 | G | A | 0.0942 | 0.0159 | 3.36E-09 |
| 93 | Atrial fibrillation | rs6462078 | A | C | 0.058 | 0.0086 | 1.35E-11 |
| 94 | Atrial fibrillation | rs62521286 | G | A | 0.1224 | 0.0148 | 1.24E-16 |
| 95 | Atrial fibrillation | rs10903345 | G | A | -0.0384 | 0.0076 | 3.98E-07 |
| 96 | Atrial fibrillation | rs28372085 | T | C | -0.093 | 0.0119 | 5.01E-15 |
| 97 | Atrial fibrillation | rs35006907 | A | C | 0.0454 | 0.0076 | 2.75E-09 |
| 98 | Atrial fibrillation | rs6993266 | A | G | 0.0443 | 0.0072 | 9.73E-10 |
| 99 | Atrial fibrillation | rs7508 | A | G | 0.072 | 0.008 | 2.22E-19 |
| 100 | Atrial fibrillation | rs4237169 | G | A | 0.0362 | 0.0074 | 1.07E-06 |
| 101 | Atrial fibrillation | rs7851198 | T | C | 0.0489 | 0.0098 | 5.90E-07 |
| 102 | Atrial fibrillation | rs4743034 | A | G | 0.049 | 0.0083 | 3.98E-09 |
| 103 | Atrial fibrillation | rs4842131 | C | T | 0.0389 | 0.0074 | 1.27E-07 |
| 104 | Atrial fibrillation | rs35991181 | C | G | -0.0419 | 0.0082 | 3.01E-07 |
| 105 | Atrial fibrillation | rs10760361 | T | G | -0.0434 | 0.0075 | 7.03E-09 |
| 106 | Atrial fibrillation | rs4385527 | A | G | 0.092 | 0.0073 | 2.26E-36 |
| 107 | Atrial fibrillation | rs4977397 | G | A | -0.0432 | 0.0075 | 8.60E-09 |
| 108 | Atrial fibrillation | rs1044258 | C | T | -0.0463 | 0.0076 | 1.07E-09 |
| 109 | Atrial fibrillation | rs71471272 | A | G | -0.0777 | 0.0158 | 8.08E-07 |
| 110 | Atrial fibrillation | rs2296610 | T | G | 0.1723 | 0.033 | 1.83E-07 |
| 111 | Atrial fibrillation | rs10745254 | G | C | 0.051 | 0.0107 | 2.01E-06 |
| 112 | Atrial fibrillation | rs7919685 | T | G | -0.0579 | 0.0071 | 5.00E-16 |
| 113 | Atrial fibrillation | rs11001667 | G | A | 0.0619 | 0.0091 | 1.06E-11 |
| 114 | Atrial fibrillation | rs11598047 | G | A | 0.1533 | 0.0095 | 4.83E-58 |
| 115 | Atrial fibrillation | rs80056983 | T | C | 0.122 | 0.0102 | 2.92E-33 |
| 116 | Atrial fibrillation | rs12571587 | C | T | -0.0345 | 0.0071 | 1.11E-06 |
| 117 | Atrial fibrillation | rs60212594 | C | G | -0.1097 | 0.0102 | 6.48E-27 |
| 118 | Atrial fibrillation | rs10906449 | C | T | -0.0337 | 0.0071 | 2.24E-06 |
| 119 | Atrial fibrillation | rs1765131 | C | G | -0.0378 | 0.0075 | 4.76E-07 |
| 120 | Atrial fibrillation | rs500523 | T | G | -0.0368 | 0.0078 | 2.38E-06 |
| 121 | Atrial fibrillation | rs76097649 | A | G | 0.1264 | 0.0137 | 2.19E-20 |
| 122 | Atrial fibrillation | rs949078 | T | C | -0.0534 | 0.0081 | 4.77E-11 |
| 123 | Atrial fibrillation | rs1822273 | A | G | -0.0683 | 0.0082 | 8.99E-17 |
| 124 | Atrial fibrillation | rs113819537 | G | C | -0.049 | 0.0082 | 2.23E-09 |
| 125 | Atrial fibrillation | rs1454933 | C | A | 0.0629 | 0.0112 | 1.76E-08 |
| 126 | Atrial fibrillation | rs883079 | T | C | 0.1196 | 0.0079 | 1.26E-51 |
| 127 | Atrial fibrillation | rs11057400 | T | C | -0.0456 | 0.0077 | 3.91E-09 |
| 128 | Atrial fibrillation | rs11180703 | A | G | -0.0457 | 0.0073 | 3.58E-10 |
| 129 | Atrial fibrillation | rs35349325 | C | T | -0.0524 | 0.0073 | 9.04E-13 |
| 130 | Atrial fibrillation | rs12810346 | T | C | 0.0658 | 0.011 | 2.34E-09 |
| 131 | Atrial fibrillation | rs10842383 | T | C | -0.1088 | 0.0104 | 1.02E-25 |
| 132 | Atrial fibrillation | rs57671871 | C | T | 0.0706 | 0.014 | 4.24E-07 |
| 133 | Atrial fibrillation | rs7978685 | C | T | -0.0547 | 0.0079 | 5.99E-12 |
| 134 | Atrial fibrillation | rs4883563 | A | G | 0.0358 | 0.0073 | 8.85E-07 |
| 135 | Atrial fibrillation | rs11835327 | G | A | 0.0681 | 0.0122 | 2.14E-08 |
| 136 | Atrial fibrillation | rs10845620 | A | G | 0.0541 | 0.0115 | 2.46E-06 |
| 137 | Atrial fibrillation | rs1323182 | G | A | 0.0398 | 0.0081 | 9.83E-07 |
| 138 | Atrial fibrillation | rs7333028 | C | T | 0.0421 | 0.0085 | 7.42E-07 |
| 139 | Atrial fibrillation | rs9580438 | C | T | 0.0568 | 0.0076 | 1.01E-13 |
| 140 | Atrial fibrillation | rs86990 | A | G | 0.04 | 0.0083 | 1.35E-06 |
| 141 | Atrial fibrillation | rs8005490 | C | T | -0.0465 | 0.0073 | 1.94E-10 |
| 142 | Atrial fibrillation | rs2145587 | A | G | 0.0754 | 0.0079 | 2.32E-21 |
| 143 | Atrial fibrillation | rs2738413 | G | A | -0.0807 | 0.0072 | 1.81E-29 |
| 144 | Atrial fibrillation | rs10873299 | G | A | -0.0483 | 0.0075 | 9.62E-11 |
| 145 | Atrial fibrillation | rs4903064 | C | T | -0.0419 | 0.0084 | 6.17E-07 |
| 146 | Atrial fibrillation | rs28631169 | T | C | 0.07 | 0.0093 | 3.80E-14 |
| 147 | Atrial fibrillation | rs12905515 | T | C | -0.0405 | 0.0088 | 4.72E-06 |
| 148 | Atrial fibrillation | rs12908437 | C | T | -0.0468 | 0.0073 | 1.25E-10 |
| 149 | Atrial fibrillation | rs12591736 | A | G | -0.0606 | 0.0102 | 2.47E-09 |
| 150 | Atrial fibrillation | rs74022964 | T | C | 0.1059 | 0.0097 | 1.27E-27 |
| 151 | Atrial fibrillation | rs1407588 | A | G | 0.0351 | 0.0074 | 2.24E-06 |
| 152 | Atrial fibrillation | rs116114803 | A | G | 0.1158 | 0.0247 | 2.71E-06 |
| 153 | Atrial fibrillation | rs2286466 | G | A | 0.0718 | 0.0095 | 3.53E-14 |
| 154 | Atrial fibrillation | rs3915425 | C | T | -0.0371 | 0.0078 | 2.12E-06 |
| 155 | Atrial fibrillation | rs11075959 | G | A | 0.1397 | 0.0216 | 1.03E-10 |
| 156 | Atrial fibrillation | rs2359171 | A | T | 0.1884 | 0.0089 | 2.94E-100 |
| 157 | Atrial fibrillation | rs8073937 | A | G | -0.0504 | 0.0074 | 1.02E-11 |
| 158 | Atrial fibrillation | rs72811294 | C | G | -0.0667 | 0.0115 | 6.87E-09 |
| 159 | Atrial fibrillation | rs12942576 | C | T | -0.0404 | 0.0078 | 2.33E-07 |
| 160 | Atrial fibrillation | rs7219869 | G | C | 0.046 | 0.0072 | 1.49E-10 |
| 161 | Atrial fibrillation | rs242557 | A | G | -0.0439 | 0.0075 | 4.35E-09 |
| 162 | Atrial fibrillation | rs2230234 | G | A | 0.07 | 0.0132 | 1.11E-07 |
| 163 | Atrial fibrillation | rs9953366 | C | T | 0.0504 | 0.0078 | 9.03E-11 |
| 164 | Atrial fibrillation | rs60318638 | C | T | 0.0433 | 0.0092 | 2.50E-06 |
| 165 | Atrial fibrillation | rs12954819 | C | G | 0.0348 | 0.0072 | 1.36E-06 |
| 166 | Atrial fibrillation | rs878767 | G | A | 0.041 | 0.0083 | 7.29E-07 |
| 167 | Atrial fibrillation | rs2656924 | C | T | 0.0579 | 0.0127 | 4.82E-06 |
| 168 | Atrial fibrillation | rs2974231 | A | G | 0.0393 | 0.0076 | 2.37E-07 |
| 169 | Atrial fibrillation | rs2145274 | C | A | -0.1015 | 0.0141 | 6.97E-13 |
| 170 | Atrial fibrillation | rs6047270 | C | T | -0.0368 | 0.0078 | 2.59E-06 |
| 171 | Atrial fibrillation | rs73272000 | A | T | 0.0634 | 0.0137 | 3.38E-06 |
| 172 | Atrial fibrillation | rs7269123 | T | C | -0.0443 | 0.0076 | 5.59E-09 |
| 173 | Atrial fibrillation | rs2834618 | G | T | -0.1096 | 0.0126 | 2.93E-18 |
| 174 | Atrial fibrillation | rs2236675 | T | C | 0.064 | 0.0128 | 5.54E-07 |
| 175 | Atrial fibrillation | rs133872 | G | C | 0.0375 | 0.0075 | 5.56E-07 |
| 176 | Atrial fibrillation | rs361834 | A | G | -0.047 | 0.0075 | 3.49E-10 |

SNP, single nucleotide polymorphism; se, standard error

**Supplementary Table 2.** The summary information for 153 SNPS selected as Mendelian stochastic variables

| **Exposure** | **SNP** | **Effect allele** | **Other allele** | **Exposure** | | | **Outcome** | | |
| --- | --- | --- | --- | --- | --- | --- | --- | --- | --- |
|  |  |  |  | **Beta** | **SE** | ***P* value** | **Beta** | **SE** | ***P* value** |
| Atrial fibrillation | rs10165883 | T | C | -0.064 | 0.007 | 5.83E-19 | -0.003 | 0.019 | 0.887 |
|  | rs10213171 | G | C | 0.104 | 0.014 | 6.09E-14 | -0.006 | 0.033 | 0.864 |
|  | rs1044258 | C | T | -0.046 | 0.008 | 1.07E-09 | -0.001 | 0.022 | 0.982 |
|  | rs10520260 | G | A | -0.054 | 0.008 | 8.98E-12 | -0.005 | 0.022 | 0.840 |
|  | rs10745254 | G | C | 0.051 | 0.011 | 2.01E-06 | -0.040 | 0.031 | 0.193 |
|  | rs10753933 | G | T | -0.074 | 0.007 | 5.83E-25 | -0.008 | 0.020 | 0.690 |
|  | rs10760361 | T | G | -0.043 | 0.008 | 7.03E-09 | 0.008 | 0.023 | 0.728 |
|  | rs10842383 | T | C | -0.109 | 0.010 | 1.02E-25 | -0.051 | 0.027 | 0.061 |
|  | rs10845620 | A | G | 0.054 | 0.012 | 2.46E-06 | 0.003 | 0.033 | 0.922 |
|  | rs10873299 | G | A | -0.048 | 0.008 | 9.62E-11 | 0.011 | 0.019 | 0.572 |
|  | rs10903345 | G | A | -0.038 | 0.008 | 3.98E-07 | -0.027 | 0.087 | 0.759 |
|  | rs10906449 | C | T | -0.034 | 0.007 | 2.24E-06 | 0.010 | 0.019 | 0.605 |
|  | rs11001667 | G | A | 0.062 | 0.009 | 1.06E-11 | -0.014 | 0.018 | 0.439 |
|  | rs11057400 | T | C | -0.046 | 0.008 | 3.91E-09 | 0.005 | 0.026 | 0.851 |
|  | rs11075959 | G | A | 0.140 | 0.022 | 1.03E-10 | -0.025 | 0.019 | 0.197 |
|  | rs11099696 | T | C | -0.038 | 0.007 | 1.37E-07 | -0.010 | 0.021 | 0.645 |
|  | rs11180703 | A | G | -0.046 | 0.007 | 3.58E-10 | 0.014 | 0.023 | 0.534 |
|  | rs11264280 | T | C | 0.127 | 0.008 | 4.60E-59 | 0.069 | 0.034 | 0.041 |
|  | rs112974895 | C | A | -0.058 | 0.012 | 4.98E-07 | 0.030 | 0.025 | 0.231 |
|  | rs113819537 | G | C | -0.049 | 0.008 | 2.23E-09 | -0.053 | 0.020 | 0.007 |
|  | rs115219487 | C | T | 0.066 | 0.013 | 1.28E-07 | 0.008 | 0.052 | 0.882 |
|  | rs11598047 | G | A | 0.153 | 0.010 | 4.83E-58 | -0.071 | 0.031 | 0.022 |
|  | rs11768850 | T | C | 0.039 | 0.007 | 4.96E-08 | -0.028 | 0.018 | 0.123 |
|  | rs11773845 | A | C | 0.116 | 0.007 | 4.61E-58 | 0.020 | 0.019 | 0.301 |
|  | rs1182197 | C | A | -0.037 | 0.007 | 3.76E-07 | 0.015 | 0.020 | 0.455 |
|  | rs11835327 | G | A | 0.068 | 0.012 | 2.14E-08 | 0.024 | 0.035 | 0.482 |
|  | rs11935444 | C | T | -0.034 | 0.007 | 3.14E-06 | -0.035 | 0.020 | 0.082 |
|  | rs12208899 | A | G | 0.049 | 0.009 | 1.95E-08 | 0.032 | 0.023 | 0.164 |
|  | rs12211255 | A | C | 0.059 | 0.012 | 2.48E-07 | 0.010 | 0.027 | 0.717 |
|  | rs12571587 | C | T | -0.035 | 0.007 | 1.11E-06 | -0.024 | 0.019 | 0.199 |
|  | rs12591736 | A | G | -0.061 | 0.010 | 2.47E-09 | 0.015 | 0.019 | 0.420 |
|  | rs12647387 | G | A | -0.067 | 0.014 | 6.14E-07 | 0.022 | 0.028 | 0.427 |
|  | rs12905515 | T | C | -0.041 | 0.009 | 4.72E-06 | -0.001 | 0.020 | 0.978 |
|  | rs12908437 | C | T | -0.047 | 0.007 | 1.25E-10 | 0.006 | 0.018 | 0.750 |
|  | rs12942576 | C | T | -0.040 | 0.008 | 2.33E-07 | 0.017 | 0.019 | 0.389 |
|  | rs12954819 | C | G | 0.035 | 0.007 | 1.36E-06 | 0.013 | 0.020 | 0.514 |
|  | rs12992412 | T | A | 0.041 | 0.007 | 2.30E-08 | -0.001 | 0.018 | 0.954 |
|  | rs1307274 | G | T | -0.074 | 0.014 | 3.85E-08 | 0.011 | 0.028 | 0.686 |
|  | rs13191450 | C | A | -0.070 | 0.008 | 8.92E-21 | 0.021 | 0.020 | 0.289 |
|  | rs1323182 | G | A | 0.040 | 0.008 | 9.83E-07 | -0.002 | 0.035 | 0.951 |
|  | rs133872 | G | C | 0.038 | 0.008 | 5.56E-07 | -0.041 | 0.025 | 0.106 |
|  | rs1407588 | A | G | 0.035 | 0.007 | 2.24E-06 | 0.017 | 0.037 | 0.651 |
|  | rs1454933 | C | A | 0.063 | 0.011 | 1.76E-08 | -0.016 | 0.026 | 0.558 |
|  | rs146518726 | A | G | 0.162 | 0.025 | 2.05E-10 | 0.156 | 0.492 | 0.751 |
|  | rs17021865 | C | T | 0.135 | 0.028 | 1.47E-06 | -0.057 | 0.062 | 0.359 |
|  | rs17042059 | A | G | 0.425 | 0.010 | 1.00E-200 | -0.043 | 0.020 | 0.030 |
|  | rs17118812 | C | T | 0.040 | 0.008 | 2.41E-07 | 0.002 | 0.019 | 0.908 |
|  | rs174048 | C | T | 0.067 | 0.010 | 1.05E-11 | -0.026 | 0.036 | 0.467 |
|  | rs17490701 | A | G | -0.070 | 0.011 | 5.43E-11 | -0.025 | 0.052 | 0.633 |
|  | rs1765131 | C | G | -0.038 | 0.008 | 4.76E-07 | 0.019 | 0.019 | 0.327 |
|  | rs179968 | T | G | -0.040 | 0.008 | 1.13E-06 | -0.005 | 0.022 | 0.816 |
|  | rs1822273 | A | G | -0.068 | 0.008 | 8.99E-17 | 0.010 | 0.018 | 0.566 |
|  | rs2044456 | G | A | 0.037 | 0.008 | 1.64E-06 | 0.011 | 0.019 | 0.561 |
|  | rs2145587 | A | G | 0.075 | 0.008 | 2.32E-21 | -0.031 | 0.019 | 0.106 |
|  | rs223369 | A | G | 0.041 | 0.008 | 6.28E-07 | 0.006 | 0.023 | 0.785 |
|  | rs2236675 | T | C | 0.064 | 0.013 | 5.54E-07 | 0.004 | 0.030 | 0.884 |
|  | rs2286466 | G | A | 0.072 | 0.010 | 3.53E-14 | 0.008 | 0.023 | 0.724 |
|  | rs2296610 | T | G | 0.172 | 0.033 | 1.83E-07 | -0.038 | 0.027 | 0.164 |
|  | rs2306272 | C | T | 0.051 | 0.008 | 4.54E-11 | -0.022 | 0.019 | 0.252 |
|  | rs2359171 | A | T | 0.188 | 0.009 | 2.94E-100 | -0.008 | 0.019 | 0.670 |
|  | rs2372992 | G | A | -0.040 | 0.008 | 3.01E-07 | -0.009 | 0.080 | 0.910 |
|  | rs242557 | A | G | -0.044 | 0.008 | 4.35E-09 | 0.006 | 0.018 | 0.751 |
|  | rs2540949 | T | A | -0.075 | 0.007 | 8.17E-25 | -0.008 | 0.019 | 0.665 |
|  | rs2656924 | C | T | 0.058 | 0.013 | 4.82E-06 | -0.010 | 0.042 | 0.821 |
|  | rs2738413 | G | A | -0.081 | 0.007 | 1.81E-29 | 0.011 | 0.019 | 0.570 |
|  | rs2834618 | G | T | -0.110 | 0.013 | 2.93E-18 | 0.056 | 0.035 | 0.113 |
|  | rs28372085 | T | C | -0.093 | 0.012 | 5.01E-15 | 0.010 | 0.048 | 0.843 |
|  | rs28631169 | T | C | 0.070 | 0.009 | 3.80E-14 | 0.007 | 0.173 | 0.968 |
|  | rs2885697 | T | G | -0.039 | 0.008 | 1.77E-07 | 0.001 | 0.019 | 0.976 |
|  | rs2949829 | T | C | -0.035 | 0.007 | 1.51E-06 | 0.005 | 0.018 | 0.795 |
|  | rs295114 | T | C | -0.068 | 0.007 | 1.76E-20 | 0.011 | 0.021 | 0.616 |
|  | rs297007 | A | C | -0.039 | 0.008 | 2.35E-06 | -0.013 | 0.021 | 0.526 |
|  | rs2974231 | A | G | 0.039 | 0.008 | 2.37E-07 | 0.008 | 0.028 | 0.776 |
|  | rs3176326 | A | G | -0.060 | 0.009 | 7.95E-11 | -0.012 | 0.030 | 0.679 |
|  | rs34118297 | T | C | 0.046 | 0.010 | 2.25E-06 | 0.001 | 0.021 | 0.962 |
|  | rs34750263 | T | C | 0.087 | 0.008 | 2.89E-30 | -0.002 | 0.019 | 0.910 |
|  | rs35006907 | A | C | 0.045 | 0.008 | 2.75E-09 | 0.034 | 0.018 | 0.061 |
|  | rs35349325 | C | T | -0.052 | 0.007 | 9.04E-13 | 0.017 | 0.025 | 0.486 |
|  | rs35504893 | T | C | 0.090 | 0.009 | 6.89E-25 | -0.014 | 0.019 | 0.474 |
|  | rs35927619 | G | T | -0.038 | 0.008 | 9.07E-07 | -0.045 | 0.029 | 0.129 |
|  | rs35991181 | C | G | -0.042 | 0.008 | 3.01E-07 | -0.016 | 0.019 | 0.376 |
|  | rs361834 | A | G | -0.047 | 0.008 | 3.49E-10 | -0.030 | 0.018 | 0.102 |
|  | rs3731326 | G | A | -0.046 | 0.008 | 6.88E-08 | -0.095 | 0.064 | 0.135 |
|  | rs3822259 | T | G | 0.046 | 0.008 | 1.93E-09 | -0.033 | 0.022 | 0.126 |
|  | rs3856795 | A | G | 0.040 | 0.008 | 1.36E-07 | 0.002 | 0.019 | 0.904 |
|  | rs3915425 | C | T | -0.037 | 0.008 | 2.12E-06 | -0.010 | 0.022 | 0.642 |
|  | rs3922843 | G | A | -0.047 | 0.008 | 8.99E-09 | -0.054 | 0.031 | 0.082 |
|  | rs4237169 | G | A | 0.036 | 0.007 | 1.07E-06 | 0.017 | 0.025 | 0.499 |
|  | rs4385527 | A | G | 0.092 | 0.007 | 2.26E-36 | 0.013 | 0.022 | 0.558 |
|  | rs4484922 | C | G | -0.063 | 0.008 | 4.57E-16 | 0.043 | 0.018 | 0.017 |
|  | rs4672423 | T | C | -0.043 | 0.007 | 5.85E-09 | -0.005 | 0.019 | 0.777 |
|  | rs4743034 | A | G | 0.049 | 0.008 | 3.98E-09 | -0.031 | 0.020 | 0.126 |
|  | rs4842131 | C | T | 0.039 | 0.007 | 1.27E-07 | 0.030 | 0.020 | 0.124 |
|  | rs4855075 | T | C | 0.060 | 0.010 | 4.00E-09 | 0.023 | 0.029 | 0.427 |
|  | rs4883563 | A | G | 0.036 | 0.007 | 8.85E-07 | 0.006 | 0.026 | 0.804 |
|  | rs4896104 | T | C | -0.035 | 0.007 | 1.79E-06 | -0.022 | 0.025 | 0.372 |
|  | rs4903064 | C | T | -0.042 | 0.008 | 6.17E-07 | 0.025 | 0.018 | 0.164 |
|  | rs4977397 | G | A | -0.043 | 0.008 | 8.60E-09 | 0.018 | 0.028 | 0.524 |
|  | rs500523 | T | G | -0.037 | 0.008 | 2.38E-06 | -0.001 | 0.021 | 0.955 |
|  | rs55734480 | A | G | 0.050 | 0.008 | 7.34E-10 | 0.029 | 0.020 | 0.147 |
|  | rs577676 | T | C | -0.096 | 0.007 | 4.38E-41 | 0.015 | 0.018 | 0.420 |
|  | rs60212594 | C | G | -0.110 | 0.010 | 6.48E-27 | -0.030 | 0.024 | 0.222 |
|  | rs60318638 | C | T | 0.043 | 0.009 | 2.50E-06 | 0.005 | 0.019 | 0.803 |
|  | rs6047270 | C | T | -0.037 | 0.008 | 2.59E-06 | 0.014 | 0.021 | 0.493 |
|  | rs62197371 | A | C | -0.040 | 0.008 | 3.65E-07 | -0.005 | 0.031 | 0.870 |
|  | rs62274627 | A | G | 0.036 | 0.008 | 1.78E-06 | -0.015 | 0.019 | 0.418 |
|  | rs62483627 | A | G | 0.049 | 0.008 | 5.17E-09 | -0.013 | 0.020 | 0.508 |
|  | rs6462078 | A | C | 0.058 | 0.009 | 1.35E-11 | 0.223 | 0.386 | 0.564 |
|  | rs6546620 | C | T | 0.071 | 0.009 | 2.96E-14 | -0.026 | 0.027 | 0.349 |
|  | rs6665642 | T | C | -0.055 | 0.012 | 4.72E-06 | -0.026 | 0.025 | 0.295 |
|  | rs6778232 | C | T | -0.041 | 0.008 | 6.29E-07 | -0.022 | 0.022 | 0.315 |
|  | rs6790396 | G | C | 0.064 | 0.007 | 4.13E-18 | -0.016 | 0.022 | 0.453 |
|  | rs6810325 | C | T | 0.075 | 0.008 | 5.24E-23 | -0.007 | 0.020 | 0.717 |
|  | rs6838973 | T | C | -0.184 | 0.007 | 1.35E-142 | 0.004 | 0.018 | 0.813 |
|  | rs6841049 | G | T | -0.037 | 0.007 | 4.88E-07 | 0.009 | 0.019 | 0.653 |
|  | rs6882776 | A | G | -0.060 | 0.008 | 3.17E-14 | 0.030 | 0.019 | 0.109 |
|  | rs6907805 | T | G | -0.041 | 0.007 | 1.10E-08 | -0.001 | 0.018 | 0.940 |
|  | rs6993266 | A | G | 0.044 | 0.007 | 9.73E-10 | -0.006 | 0.020 | 0.764 |
|  | rs716845 | A | G | 0.059 | 0.008 | 1.16E-13 | 0.014 | 0.026 | 0.600 |
|  | rs7219869 | G | C | 0.046 | 0.007 | 1.49E-10 | 0.016 | 0.019 | 0.397 |
|  | rs7269123 | T | C | -0.044 | 0.008 | 5.59E-09 | -0.016 | 0.019 | 0.393 |
|  | rs72811294 | C | G | -0.067 | 0.012 | 6.87E-09 | -0.040 | 0.033 | 0.220 |
|  | rs73032363 | G | A | -0.043 | 0.008 | 3.59E-08 | -0.001 | 0.020 | 0.967 |
|  | rs73206619 | T | G | 0.041 | 0.008 | 8.43E-07 | -0.013 | 0.018 | 0.484 |
|  | rs73272000 | A | T | 0.063 | 0.014 | 3.38E-06 | 0.030 | 0.046 | 0.512 |
|  | rs7333028 | C | T | 0.042 | 0.009 | 7.42E-07 | 0.037 | 0.030 | 0.224 |
|  | rs74022964 | T | C | 0.106 | 0.010 | 1.27E-27 | 0.008 | 0.054 | 0.888 |
|  | rs7508 | A | G | 0.072 | 0.008 | 2.22E-19 | -0.012 | 0.018 | 0.499 |
|  | rs7549338 | G | C | -0.045 | 0.007 | 1.71E-10 | 0.024 | 0.018 | 0.179 |
|  | rs7618629 | G | A | -0.038 | 0.008 | 3.03E-06 | -0.016 | 0.019 | 0.388 |
|  | rs7632427 | C | T | -0.043 | 0.007 | 1.10E-08 | -0.010 | 0.028 | 0.726 |
|  | rs7719528 | T | C | -0.036 | 0.007 | 6.67E-07 | 0.029 | 0.020 | 0.141 |
|  | rs7789146 | A | G | -0.057 | 0.009 | 6.51E-10 | 0.029 | 0.021 | 0.158 |
|  | rs7851198 | T | C | 0.049 | 0.010 | 5.90E-07 | -0.010 | 0.044 | 0.826 |
|  | rs78758741 | C | T | 0.061 | 0.012 | 4.79E-07 | -0.011 | 0.053 | 0.829 |
|  | rs79187193 | A | G | -0.112 | 0.018 | 8.07E-10 | -0.105 | 0.114 | 0.358 |
|  | rs7919685 | T | G | -0.058 | 0.007 | 5.00E-16 | -0.013 | 0.019 | 0.487 |
|  | rs7978685 | C | T | -0.055 | 0.008 | 5.99E-12 | -0.001 | 0.019 | 0.972 |
|  | rs79835095 | T | C | 0.045 | 0.009 | 2.03E-07 | -0.035 | 0.028 | 0.213 |
|  | rs8005490 | C | T | -0.047 | 0.007 | 1.94E-10 | 0.019 | 0.022 | 0.394 |
|  | rs80056983 | T | C | 0.122 | 0.010 | 2.92E-33 | -0.038 | 0.019 | 0.052 |
|  | rs8073937 | A | G | -0.050 | 0.007 | 1.02E-11 | -0.005 | 0.018 | 0.801 |
|  | rs86990 | A | G | 0.040 | 0.008 | 1.35E-06 | -0.015 | 0.024 | 0.536 |
|  | rs878767 | G | A | 0.041 | 0.008 | 7.29E-07 | 0.046 | 0.046 | 0.324 |
|  | rs880315 | C | T | 0.044 | 0.008 | 5.04E-09 | -0.014 | 0.019 | 0.480 |
|  | rs883079 | T | C | 0.120 | 0.008 | 1.26E-51 | -0.021 | 0.018 | 0.253 |
|  | rs9481842 | G | T | 0.066 | 0.008 | 8.12E-16 | -0.038 | 0.037 | 0.305 |
|  | rs949078 | T | C | -0.053 | 0.008 | 4.77E-11 | 0.035 | 0.019 | 0.073 |
|  | rs9580438 | C | T | 0.057 | 0.008 | 1.01E-13 | -0.015 | 0.024 | 0.524 |
|  | rs976568 | T | G | -0.139 | 0.008 | 4.54E-76 | 0.018 | 0.019 | 0.338 |
|  | rs9862378 | T | G | 0.033 | 0.007 | 4.25E-06 | -0.041 | 0.021 | 0.054 |
|  | rs9872035 | T | C | -0.037 | 0.007 | 1.63E-07 | 0.011 | 0.020 | 0.579 |
|  | rs9953366 | C | T | 0.050 | 0.008 | 9.03E-11 | 0.009 | 0.018 | 0.607 |

**Supplementary Figure S2 Forest plot (A), sensitivity analysis (B), scatter plot (C) and funnel plot (D) of the causal effect of Atrial fibrillation on Chronic gastritis risk.**

**Supplementary Figure S3 Forest plot (A), sensitivity analysis (B), scatter plot (C) and funnel plot (D) of the causal effect of Atrial fibrillation on Helicobacter pylori risk.**


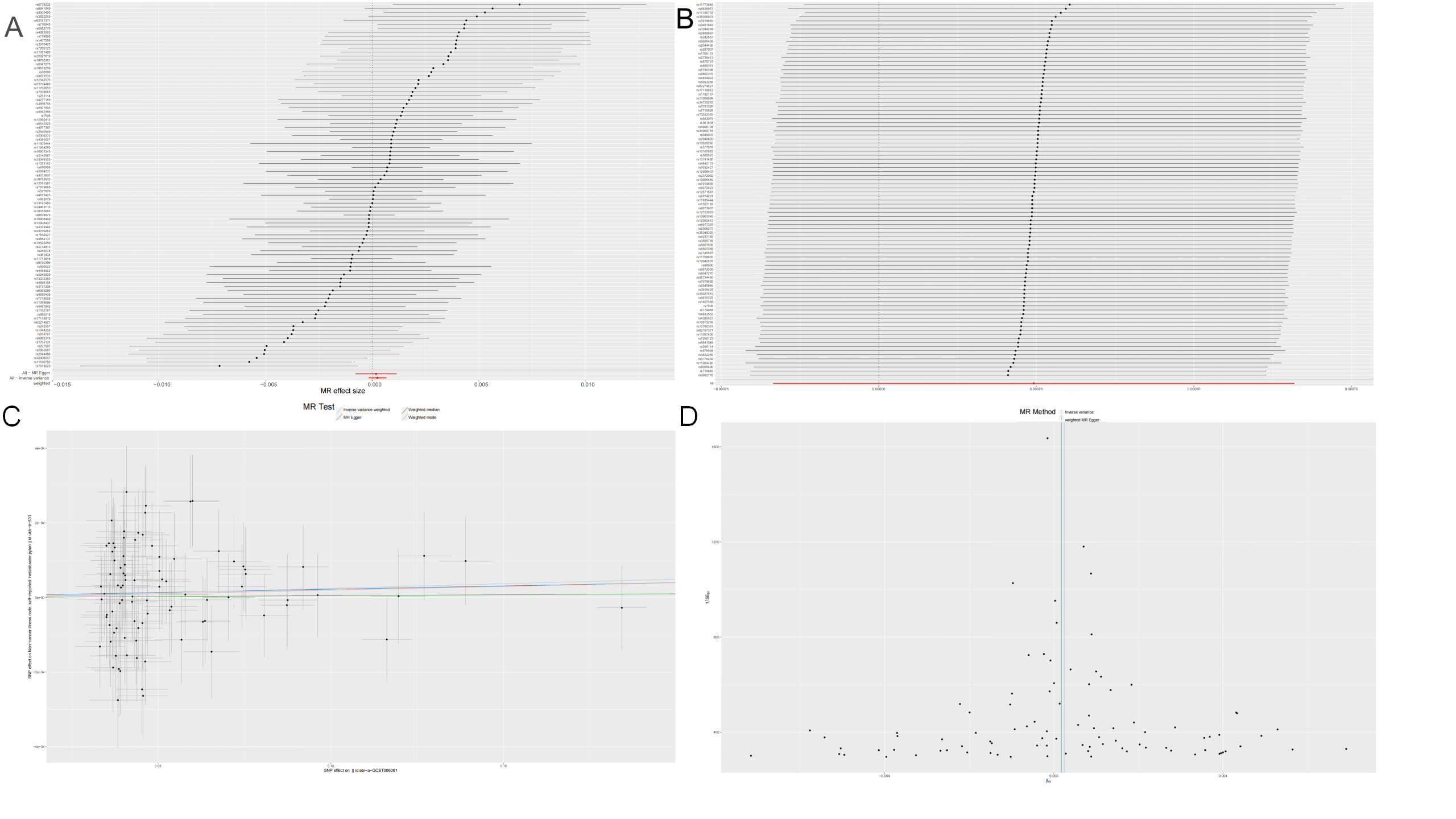


**Supplementary Figure S4 Forest plot (A), sensitivity analysis (B), scatter plot (C) and funnel plot (D) of the causal effect of Atrial fibrillation on Alcohol drinker status: Current.**


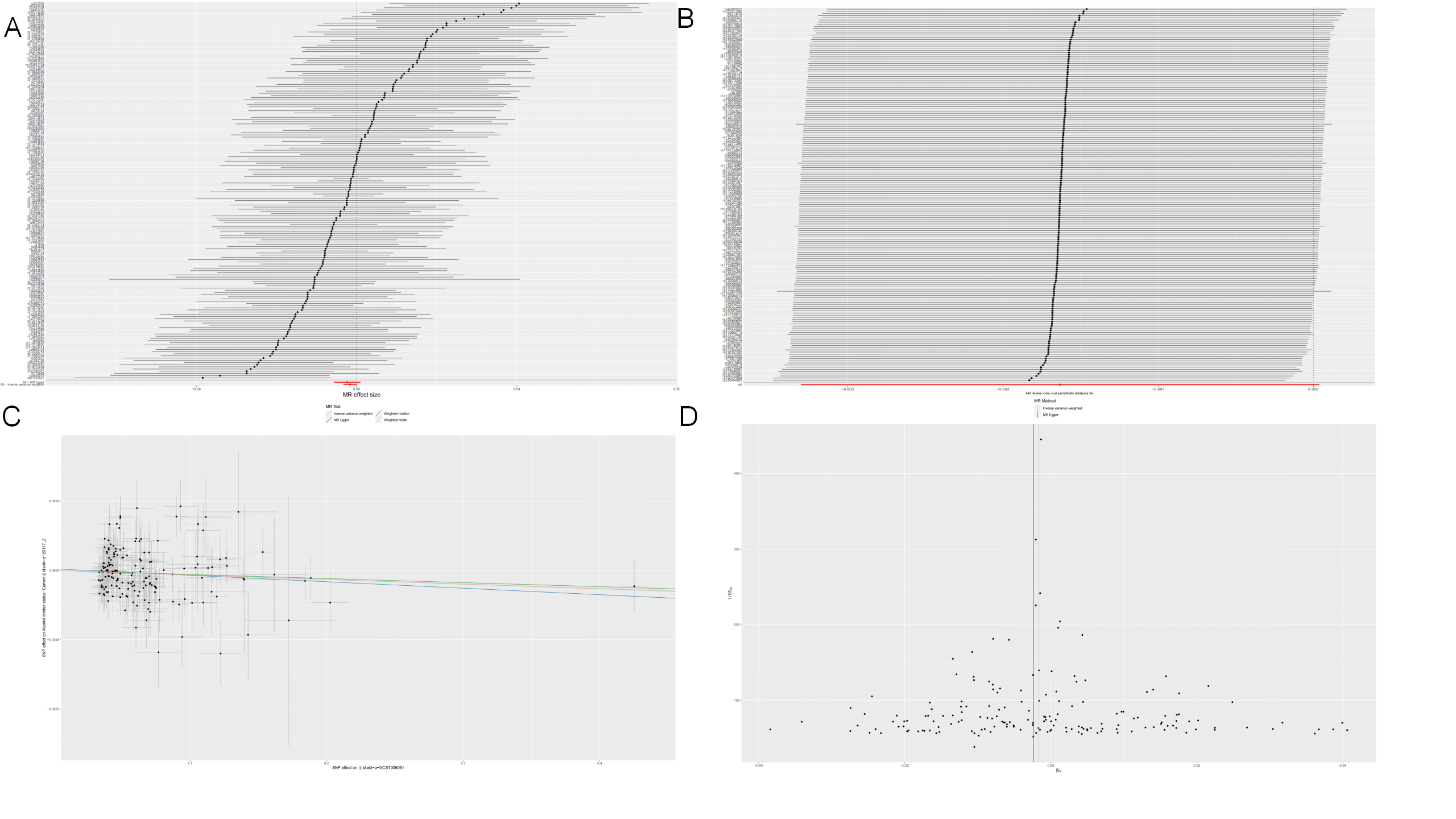


**Supplementary Figure S5 KM survival graphs of patients at different risk based on prognostic models in the GSE62254 dataset**


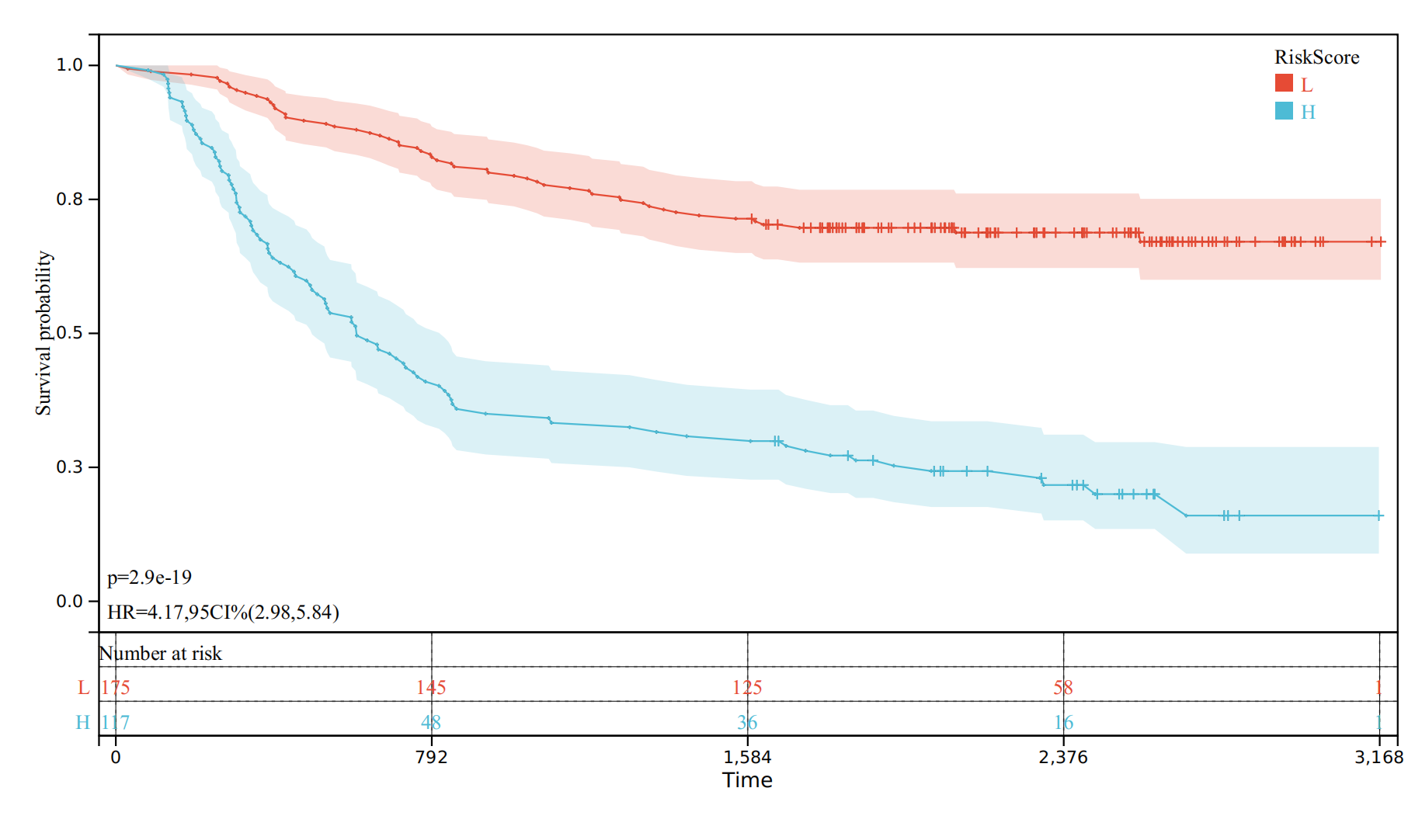


**Supplementary Figure S6 ROC curves for 1-year,3-year, and 5-year survival of patients were predicted based on prognostic models in the GSE62254 dataset.**


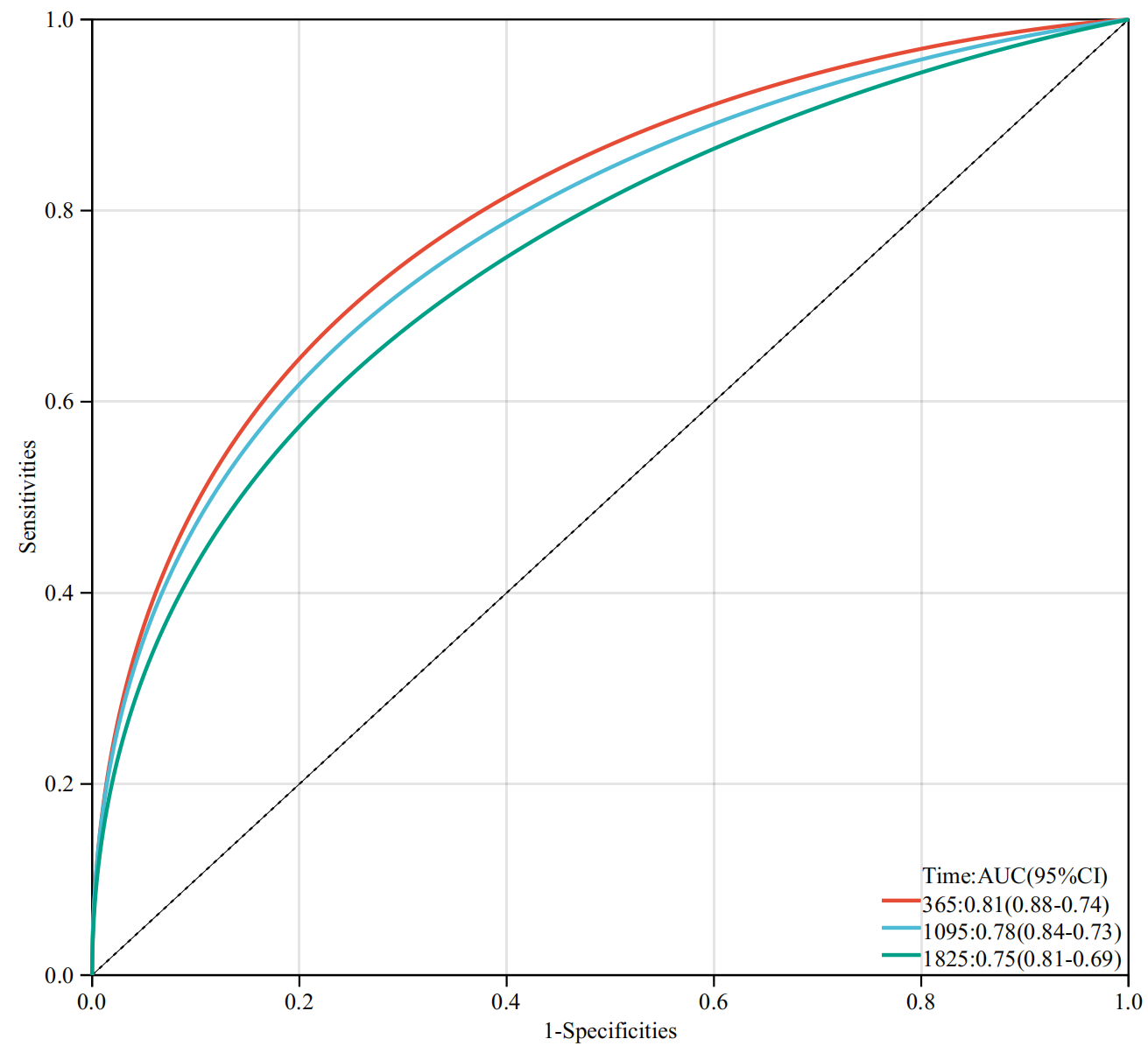

Supplement: Supplementary file 1 [file Table1.docx]
